# Glutaredoxin‐1 promotes lymphangioleiomyomatosis progression through inhibiting Bim‐mediated apoptosis via COX2/PGE2/ERK pathway

**DOI:** 10.1002/ctm2.1333

**Published:** 2023-07-21

**Authors:** Ya Feng, Tianjiao Li, Yin Li, Zhoujun Lin, Xiao Han, Xiaolin Pei, Yupeng Zhang, Fei Li, Juan Yang, Di Shao, Chenggang Li

**Affiliations:** ^1^ State Key Laboratory of Medicinal Chemical Biology and College of Pharmacy Nankai University Tianjin P. R. China; ^2^ Chongqing University Central Hospital Chongqing Emergency Medical Center Chongqing P. R. China

**Keywords:** apoptosis, Bim, glutaredoxin, lymphangioleiomyomatosis, oxidative stress

## Abstract

**Background:**

Lymphangioleiomyomatosis (LAM) is a female‐predominant interstitial lung disease, characterized by progressive cyst formation and respiratory failure. Clinical treatment with the mTORC1 inhibitor rapamycin could relieve partially the respiratory symptoms, but not curative. It is urgent to illustrate the fundamental mechanisms of TSC2 deficiency to the development of LAM, especially mTORC1‐independent mechanisms. Glutaredoxin‐1 (Glrx), an essential glutathione (GSH)‐dependent thiol‐oxidoreductase, maintains redox homeostasis and participates in various processes via controlling protein GSH adducts. Redox signalling through protein GSH adducts in LAM remains largely elusive. Here, we demonstrate the underlying mechanism of Glrx in the pathogenesis of LAM.

**Methods:**

1. Abnormal Glrx expression in various kinds of human malignancies was identified by the GEPIA tumour database, and the expression of Glrx in LAM‐derived cells was detected by real‐time quantitative reverse transcription (RT‐qPCR) and immunoblot. 2. Stable Glrx knockdown cell line was established to evaluate cellular impact. 3. Cell viability was determined by CCK8 assay. 4. Apoptotic cell number and intracellular reactive oxygen species (ROS) level were quantified by flow cytometry. 5. Cox2 expression and PGE2 production were detected to clarify the mechanism of Bim expression modulated by Glrx. 6. S‐glutathionylated p65 was enriched and detected by immunoprecipitation and the direct regulation of Glrx on p65 was determined. 7. The xenograft animal model was established and photon flux was analyzed using IVIS Spectrum.

**Results:**

In LAM, *TSC2* negatively regulated abnormal Glrx expression and activation in a mTORC1‐independent manner. Knockdown of Glrx increased the expression of Bim and the accumulation of ROS, together with elevated S‐glutathionylated proteins, contributing to the induction of apoptotic cell death and inhibited cell proliferation. Knockdown of Glrx in TSC2‐deficient LAM cells increased GSH adducts on nuclear factor‐kappa B p65, which contributed to a decrease in the expression of Cox2 and the biosynthesis of PGE2. Inhibition of PGE2 metabolism attenuated phosphorylation of ERK, which led to the accumulation of Bim, due to the imbalance of its phosphorylation and proteasome degradation. In xenograft tumour models, knockdown of *Glrx* in TSC2‐deficient LAM cells inhibited tumour growth and increased tumour cell apoptosis.

**Conclusions:**

Collectively, we provide a novel redox‐dependent mechanism in the pathogenesis of LAM and propose that Glrx may be a beneficial strategy for the treatment of LAM or other TSC‐related diseases.

## Graphical abstract

- TSC2 negatively regulates Glutaredoxin‐1 (Glrx) hyperactivation in a mTORC1‐independent manner in lymphangioleiomyomatosis (LAM).
- Abnormal Glrx inhibits reactive oxygen species accumulation and decreases glutathione adducts on nuclear factor‐kappa B p65, which contributed to promoting Cox2 expression and the biosynthesis of PGE2.
- Increased PGE2 metabolism exacerbates phosphorylation of ERK, which leads to proteasome degradation of Bim and further induces cell survival in LAM.

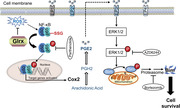

## 1 INTRODUCTION

Lymphangioleiomyomatosis (LAM) is a rare lung disease of unknown aetiology, primarily affecting young women. The proposed pathogenesis of LAM holds that histologically characterized by a diffuse proliferation of atypical smooth muscle cells (LAM cells) in the alveoli and cystic degeneration of the normal lung parenchyma, which leads to cystic parenchymal destruction and progressive respiratory failure.^1^, ^2^ LAM occurs sporadically (S‐LAM) or with germline TSC1 or TSC2 mutations (TSC‐LAM).^3^, ^4^ These mutations lead to subsequently abnormal activation of the mammalian target of Rapamycin (mTOR) signalling and elevate LAM cell proliferation.^5^, ^6^ This understanding results in multiple preclinical studies about the target of mTOR. mTORC1 inhibitors, such as Rapamycin analogues, are currently used as the first‐line therapy to treat LAM.^7^ However, Rapamycin relieves partially the respiratory symptoms but is not curative. Therefore, the development of new therapeutic targets is still urgently required.

Protein glutathione (GSH) adducts (referred to as S‐glutathionylation), a reversible oxidative post‐translational modification (OPTM) of protein cysteines involved in redox signalling, have been recognized for their critical roles in physiology and pathophysiology.^8^, ^9^ Protein cysteines can be reversibly oxidized to sulfenic acid (R‐SOH) or form disulfide bonds (R‐S‐S‐R) or form reversible S‐nitrosothiols (R‐SNO), which can be finally reversibly modified by forming an S‐glutathionylation.^10^ If the redox homeostasis is disrupted, the protein cysteine residues are modified and form sulfinic (R‐SO_2_H) and irreversible sulfonic acid (R‐SO_3_H), which can induce permanent changes in protein structure and function.^11^, ^12^, ^13^ Importantly, in recent years, mounting pieces of evidence suggest that GSH adducts, controlled by glutaredoxins (Glrxs) as a redox switch, participate in various human diseases including cancer to cardiovascular diseases.^14^, ^15^

Glrxs are essential glutathione (GSH)‐dependent thioltransferases and display a general thiol‐disulfide oxidoreductase activity.^16^ In humans, GLRXs have been identified as two dithiol isoforms (i.e. GLRX1 (Glrx) and GLRX2) and one monothiol isoform (i.e. GLRX5).^17^ Notably, Glrx is the most efficient at deglutathionylating proteins, which catalyzes the reduction of GSH adducts and confers reversible signalling function to proteins with redox‐sensitive thiols.^18^, ^19^ Recent studies have demonstrated that GSH adducts, controlled by Glrx, participate in the regulation of many cellular processes, including proliferation,^20^ apoptosis,^21^, ^22^ lung fibrosis,^23^ angiogenesis,^24^ inflammation^25^, ^26^ and metabolism.^27^ In this study, we observed abnormal Glrx expression, accompanied by differentiated protein GSH adducts in TSC2‐deficient cells. This implies that Glrx may be the key regulator in the progression of LAM.

We explored the underlying mechanism of Glrx in the pathogenesis of LAM. Our data suggested that Rapamycin‐independently hyperactivation of Glrx in LAM attenuated intracellular oxidative stress and Bim‐mediated apoptosis, which is related to decreased GSH adducts on p65 nuclear factor‐kappa B (NF‐κB). Collectively, our findings provided new insights into the pathogenesis of LAM and supported a novel appealing therapeutic target in LAM treatment.

## 2 MATERIALS AND METHODS

### 2.1 Cell lines

Eker rat uterine leiomyoma‐derived TSC2‐deficient ELT3‐V3 cells and TSC2‐addback ELT3‐T3 cells were provided by C. Walker, Institute of Biosciences and Technology, Texas A&M University, Houston, USA. LAM‐derived cells (including TSC2‐deficient 621‐101 cells and TSC2‐addback 621‐103 cells) were provided by Harvard Medical School. All cell lines were cultured in 5% CO_2_ at 37°C with the condition of Dulbecco's modified Eagle's medium (DMEM) containing 10% fetal bovine serum (FBS; Hyclone) and 1% penicillin‐streptomycin.

### 2.2 Reagents and antibodies

Rapamycin (ENZO), Torin1 (cayman), AZD6244 (cayman), Parthenolide (cayman), Bortezomib (cayman), prostaglandin E2 (MCE). Antibodies used were Anti‐glutaredoxin‐1 (Abcam, catalogue ab45953); Bim (ENZO, catalogue ADI‐AAP‐330‐E); β‐actin (EASYBIO, catalogue BE0037); Anti‐Glutathione (Santa Cruz, catalogue sc‐52399); p65 (Santa Cruz, catalogue sc‐8008); Bim (Santa Cruz, catalogue sc‐374358). The antibodies below are from Cell Signaling Technology: Tuberin (catalogue 4308); phospho‐Akt (S473) (catalogue 9271); phospho‐S6(Ser235/236) (catalogue 2211); Raptor (catalogue 2280); Rictor (catalogue 2114); cleaved caspase3 (catalogue 9661); cleaved PARP (catalogue 9546); Cox2 (catalogue 12282); phospho‐p65(Ser536) (catalogue 3033); phospho‐ERK1/2(T202/Y204) (catalogue 9101).

### 2.3 Lentivirus production and transfection

The sh*Glrx*, sh*COX2*, sh*Raptor*, sh*Rictor* or scrambled shRNA plasmids (designed by GenePharma, China) were transfected into HEK‐293T cells to produce lentivirus particles. For stable transfection, 621‐101 cells were incubated for 24 h in a culture medium enriched in retroviral particles. Then, the supernatant was discarded and then added fresh medium with 10 μg/mL puromycin. Following 2 weeks of puromycin selection, single colonies were obtained. The cells were transiently transfected with plasmids overexpressing *Glrx*, *TSC2 BIM* and an empty vector using the Lipofectamine 2000 (Invitrogen).

### 2.4 Immunoblot assay

The radioimmunoprecipitation assay (RIPA) lysis buffer was used to extract protein. The equal amounts of protein were separated by SDS‐PAGE and then transferred onto polyvinylidene fluoride membranes. The membrane was blocked with 3% BSA for 1 h and then incubated with specific primary antibodies overnight at 4°C. On day 2, the membrane was washed with TBST and then incubated with specific secondary antibodies for 1 h.

### 2.5 RNA extraction and RT‐qPCR

TRIzol regent (Ambion, LOT No.317110) was used for total RNA isolation. Reverse transcriptase (YEASEN, catalogue No.11141ES60) was used to reverse‐transcribe RNA into cDNA. 500 ng of cDNA as the template was performed for real‐time quantitative reverse transcription (RT‐qPCR) assay. The gene‐specific primer sequences (AuGCT DNA‐SYN Biotechnology) were designed (Table S1). The SuperReal PreMix Plus (SYBR Green) regent (TIANGEN, catalogue No.#FP205‐02) was used to perform amplification reactions of RT‐qPCR following as 95°C for 15 min, then followed by three‐step amplification cycles as a denaturation for 10 s at 95°C, annealing for 20 s at 55°C and extension for 30 s at 72°C.

### 2.6 Cell viability assay

Glrx‐depleted or control 621‐101 cells with the same density (3000 cells/well) were plated in a 96‐well plate overnight. On day 2, the old medium was discarded and the fresh DMEM medium with 2% FBS was added. At the indicated time, the CCK8 reagent was added and incubated for 2 h. The OD_450 nm_ was read by the enzyme labelling instrument.

### 2.7 EdU proliferation assay

The cells were seeded in a plate overnight, then incubated for 3 h with 10 μM of EdU (5‐ethynyl‐2′‐deoxyuridine). As per the instruction of the EdU‐488 assay kit (Beyotime, catalogue C0071S), cells were fixed for 15 min using 4% formaldehyde and followed by permeabilization for 10 min using 0.3% Triton X‐100. The Confocal laser scanning microscope or flow cytometry (BD LSRFortessa) was applied for making the quantification of EdU‐positive cells.

### 2.8 Cell apoptosis assay

Glrx‐depleted or control 621‐101 cells were seeded and incubated overnight. On day 2, the fresh DMEM medium with 1%FBS was added for 48 h treatment. Cell pellets were collected and then followed by resuspending in 300 μl binding buffer. As per the instruction of the apoptosis kit (MultiSciences, catalogue 70‐AP105‐100), 3 μl of APC conjugated Annexin V and 6 μl of 7‐AAD were mixed with cell suspension for 15 min. The stained Annexin V‐positive cells were quantified by flow cytometry.

### 2.9 Cell cycle assay

621‐101 sh*NC* and sh*Glrx* cells were collected and washed twice. Then Regent A and B from Cell Cycle Staining Kit (MultiSciences, CCS012) were mixed with cells and incubated for 30 min. Using flow cytometry to analyze cell cycle distribution.

### 2.10 Immunohistochemistry

5 μm thick section on tissue slides were deparaffinized three times in xylene, then followed by rehydration in serial ethanol. Sections were permeabilized by 0.5% Triton X‐100 and quenched for peroxidase by 3% H_2_O_2_. Following antigen retrieval for 30 min and blocking with 10% goat serum, Immunostaining was performed with specific antibodies incubation. On day 2, with specific secondary antibody incubation for 1 h, the DAB detection system (Solarbio, SW1020) and Hematoxylin (Solarbio, H8070) were used for analysis.

### 2.11 Immunofluorescence staining

Cells were seeded and incubated overnight. On day 2, the fresh medium containing 1% FBS was added. After 48 h, the cells were fixed and then permeated by 0.2% Triton X‐100. After blocking, the primary antibody was added to the well with incubation overnight at 4°C. On day 2, Alexa Fluor 594 or FITC‐conjugated secondary antibodies were added with incubation for 1 h. Following DAPI staining of the nucleus, representative images were captured. Similarly, the xenograft tumour tissues performed Immunofluorescence staining as previously described.

### 2.12 Intracellular reactive oxygen species detection

The reactive oxygen species (ROS) level was analyzed using the kit (Solarbio, CA1410). 621‐101 cells with Glrx‐depletion or control were plated in 6‐well cell culture plates overnight. Then DMEM medium with 1% FBS was added. After 24 h. DCFH‐DA was loaded for 30 min at 37°C and then measure the fluorescence.

### 2.13 Detection of mitochondrial membrane potential

Mitochondrial membrane potential (MMP) was measured by Mitochondria Staining Kit (MultiSciences, 70‐MJ101). Glrx‐depleted or control 621‐101 cells were cultured in DMEM with 1% FBS. After 48 h, JC‐1 was loaded (2 μM) and the fluorescence was analyzed by flow cytometry setting as an excitation at 488 nm and emissions at 530 nm for green and 590 nm for red fluorescence. MMP was evaluated using the percentage of fluorescence ratio (590/530 nm).

### 2.14 Quantification of prostaglandin E2

Glrx‐depleted 621‐101 cells or Glrx‐overexpressed 621‐103 cells were plated in a 6‐well plate for 48 h. Then the supernatants of cell culture were collected. The level of secreted PGE2 was measured using an ELISA kit (MultiSciences, catalogue EK8103/2‐0). The relative level of secreted PGE2 was shown as a fold of change by normalizing to control cells.

### 2.15 Quantification of GSH

Collect cell pellets and lysis them by repeated freeze‐thawing in liquid nitrogen. The samples from each group were adjusted to the same concentration by BCA protein quantification. The total levels of GSH in cells were quantified using the GSH Assay Kit (Beyotime, catalogue S0053). Then relative GSH levels were shown by normalizing to control cells.

### 2.16 Immunoprecipitation assay

Collect and lysis cell pellets for 30 min using nondenaturing RIPA lysis buffer (Containing 1 M Tris‐Hcl pH 7.4, Nacl, TritonX‐100, Sodium deoxycholate, 10%SDS) supplemented with phosphatase and protease inhibitor cocktails (Thermo Fisher Scientific, 78440) and 20 mM N‐ethylmaleimide (MCE, 128‐53‐0), then followed by protein quantifications using BCA kit. Next, equal amounts of cell lysates were immunoprecipitated with anti‐GSH antibodies using rProtein A/G MagBeads (YEASEN, 36417ES03). After washing beads, Immunoblot was performed to analyze the p65 level from Immunoprecipitates. The specific steps were done according to the instructions on the kit.

### 2.17 Chromatin immunoprecipitation assay

Chromatin immunoprecipitation (ChIP) assay was performed using the kit (Absin, abs50034). Using p‐p65 antibody to pull down complexes of DNA and protein. The primers for the region of the p65 binding motif (nucleotides −1915 to −1532) in Cox2 promoter sequences were designed as 5′‐ACTCTTCTCGCTCCGCTTTC‐3′ and 5′‐GCTGCCTCAGTTTCCCTATCT‐3′, with an expected size of 384 bp.

### 2.18 Colony formation assay

Cells with a density of 2–3×10^3^ cells/well were seeded. 621‐103 cells were transiently transfected with Glrx overexpressed plasmids. Then change to fresh medium containing 2% FBS every third day. After 14 days, cells were stained with 0.05% crystal violet and representative images were captured.

### 2.19 Xenograft tumour model

Female BALB/c mice at 6 to 8 weeks of age were randomly divided into two groups. Equal numbers (6 × 10^6^ cells) of ELT3 cells (ELT3‐T3 and ELT3‐V3 cells) were injected in both flanks subcutaneously (*n* = 3 for each group). After 40 days, the tumours were removed. For the 621‐101 xenograft tumour model, equal numbers (5×10^6^ cells mixed with 100 μl matrigel) of luciferase‐expressing 621‐101 sh*NC* or sh*Glrx* cells were injected into BALB/c female mice subcutaneously. Tumor volumes were monitored using the formula as Length × Width^2^/2 and bioluminescent imaging was captured. The growth of the tumour was slow, taking 8 weeks until palpable tumour nodules appeared. After 11 weeks, tumours were removed.

### 2.20 Bioluminescent imaging of intravenous tumour model

100 μl of PBS including Luciferase‐expressing 621‐101sh*NC* or sh*Glrx* Cells (5×10^5^cells) was injected intravenously into BALB/c female mice. At 0, 3, 9 and 24 h after cells injection, mice were given D‐luciferin (PerkinElmer Inc, catalogue 122799) by Intraperitoneal Injections according to 120 mg/kg dose. And using IVIS Spectrum System to capture bioluminescent images and read the total photon flux of chest regions.

### 2.21 TUNEL assay

The tissues from 621‐101 xenograft tumours were embedded using paraffin, then followed by cutting into sections and staining. Apoptotic cells were analyzed using the TUNEL Apoptosis Detection Kit (FITC) (ABSIN, abs50033). The confocal laser scanning microscope was used to capture representative images.

### 2.22 Statistical analysis

Using a student's *t*‐test to compare differences between groups by GraphPad Prism 8.0 software. All data were represented as the mean ± SEM. *p* < .05 was considered statistically significant.

## 3 RESULTS

### 3.1 Abnormal Glrx expression was identified in TSC2‐deficient cells in vitro and in vivo

**FIGURE 1:**
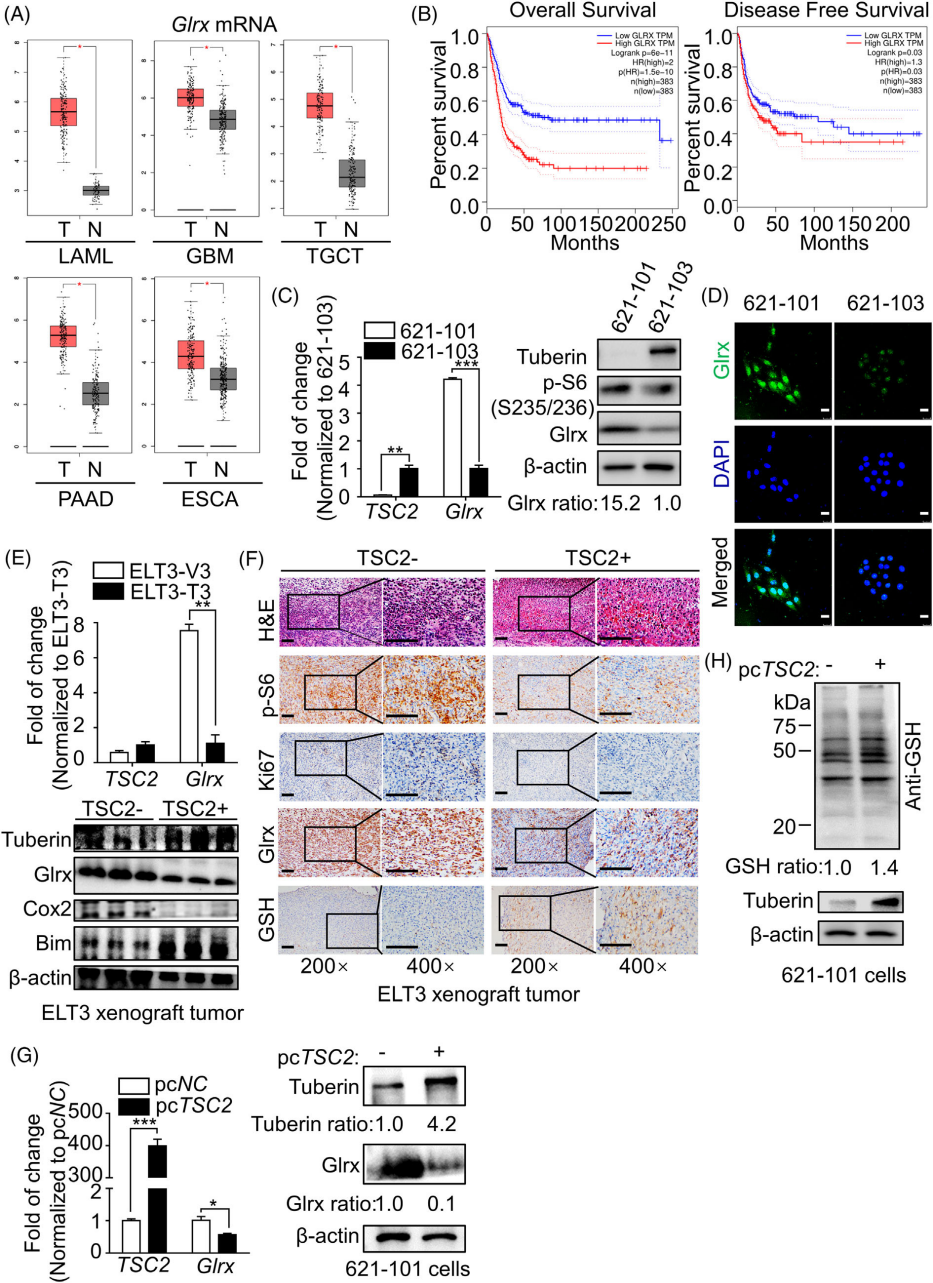
Abnormal glutaredoxin‐1 (Glrx) was identified in TSC2‐deficient cells in vitro and in vivo. (A) Box plots of *Glrx* mRNA levels in several different types of tumour samples (T, red) and normal samples (N, gray). Database: GEPIA. (B) GEPIA analysis of the correlation between Glrx expression and the overall survival rate (OS) and the disease‐free survival (DFS) rate among patients with several different types of cancers (*p* < .05, log‐rank test). (C) RT‐qPCR analysis (left) of *TSC2* and *Glrx*, and Immunoblot analysis (right) of Tuberin, p‐S6(S235/236), Glrx in lymphangioleiomyomatosis (LAM)‐derived TSC2‐deficient 621‐101 cells and TSC2‐addback 621‐103 cells. (D) Representative images of Glrx expression detected by Immunofluorescence in LAM‐derived cells (621‐101 and 621‐103 cells). DAPI was used for nucleus staining. Scale Bar, 25 μm. (E) RT‐qPCR analysis (upper) of *TSC2* and *Glrx*, and the levels of Tuberin, Glrx, Cox2 and Bim in ELT3 xenograft tumors determined by Immunoblot assay. (F) H&E staining (upper) and Immunohistochemical staining (lower) of p‐S6(S235/236), Ki67, Glrx and glutathione (GSH) in ELT3 xenograft tumour tissues. magnification ×200 or ×400. Scale Bar, 100 μm. (G) RT‐qPCR and Immunoblot analysis of the levels of TSC2 and Glrx in 621‐101 cells transfected with *TSC2*‐overexpressed plasmid for 72 h. (H) The levels of protein GSH adducts were detected by Immunoblot assay in 621‐101 cells transfected with *TSC2*‐overexpressed plasmid for 72 h. Student's t‐test, *p < .05, **p < .01, ***p < .001.

Glrx is a necessary thioltransferase to regulate reversible oxidative modification of proteins. GEPIA dataset^28^ demonstrated an abnormal *Glrx* expression in various kinds of human malignancies, including acute myeloid leukaemia, glioblastoma multiforme, pancreatic adenocarcinoma, testicular germ cell tumours and oesophagal carcinoma, compared with healthy people (normal) (Figure 1A). In accordance with previous studies that the increased Glrx was closely associated with poor prognosis in certain cancers,^29^, ^30^ GEPIA survival analysis showed that higher Glrx expression conferred poor overall survival (OS) and disease‐free survival (DFS) (*p* < .05) in certain cancers (Figure 1B). Furthermore, GEPIA correlation analysis showed a negative correlation between *TSC2* and *Glrx* in cancers (Figure S1A), suggesting a potential contribution of Glrx in the development of LAM.

Based on these findings, Immunoblot and RT‐qPCR analyses were performed in LAM. Compared with 621‐103 cells, abnormal Glrx expression was observed in 621‐101 cells, accompanied by p‐S6(S235/236) hyperactivation, a downstream effector of mTORC1 (Figure 1C). Subsequently, Immunofluorescence analysis also confirmed abnormal Glrx expression and differentiated protein GSH adducts in 621‐101 cells (Figure 1D and Figure S1C). To eliminate tissue specificity, we further analyzed the expression of Glrx in rat uterus–derived ELT3 cells. As expected, Immunoblot and RT‐qPCR analysis showed that Glrx was highly expressed in ELT3‐V3 cells compared with ELT3‐T3 cells (Figure S1D), which was consistent with the results in vivo confirmed by Immunoblot and RT‐qPCR analysis (Figure 1E). Similarly, H&E and Immunohistochemical staining analysis demonstrated that the expressions of Glrx, p‐S6(S235/236) and Ki67 (the cell proliferation marker) significantly increased and the level of protein GSH adducts significantly decreased, corresponding to overexpression of Glrx in ELT3‐V3 xenografts (Figure 1F). These results revealed that Glrx expression was generally increased in TSC2‐deficient cells, both in vitro and in vivo.

**FIGURE 2:**
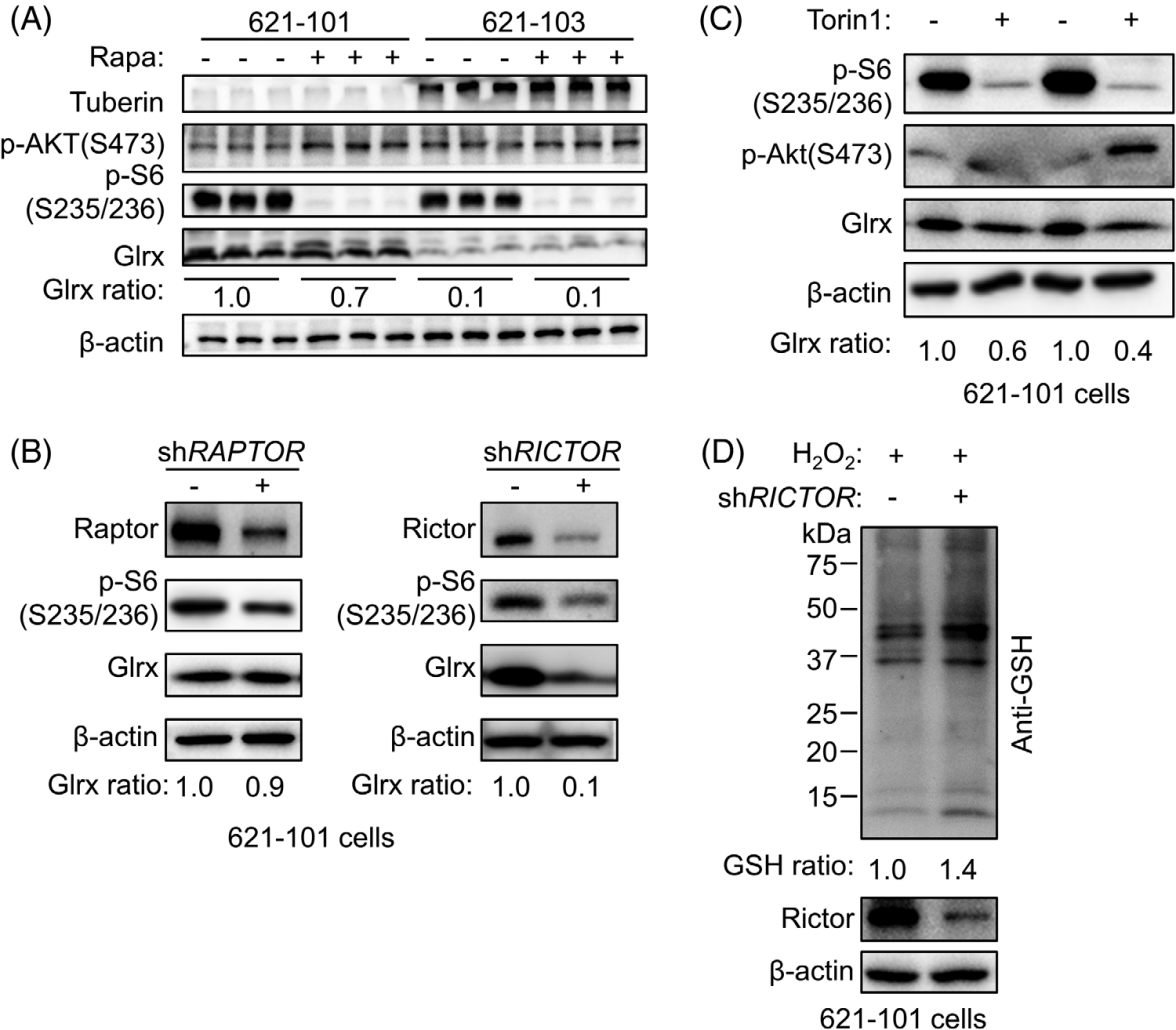
TSC2 negatively regulated glutaredoxin‐1 (Glrx) expression in a rapamycin‐insensitive manner in vitro. (A) Immunoblot analysis of Tuberin, p‐AKT(S473), p‐S6(S235/236) and Glrx in 621‐101 cells treated with 20 nM rapamycin for 24 h. (B) Immunoblot analysis of p‐S6(S235/236), Glrx, Raptor or Rictor in 621‐101 cells after transfection with *RAPTOR* (left) or *RICTOR* (right) shRNA. (C) Immunoblot analysis of p‐S6(S235/236), p‐AKT (S473) and Glrx in 621‐101 cells treated with 250 nM Torin1 for 24 h. (D) The levels of protein glutathione (GSH) adducts were assessed by Immunoblot in 621‐101 cells transfected with control or *RICTOR* shRNA with 300 μM H_2_O_2_ stimulation.

To investigate whether the TSC2/mTOR signalling is involved in regulating the expression of Glrx, Immunoblot and RT‐qPCR assays were performed and we found that overexpression of TSC2 decreased Glrx expression in 621‐101 cells (Figure 1G), together with elevated protein GSH adducts (Figure 1H). In addition, we also observed upregulated Glrx expression in *TSC2* knockdown 293T cells (Figure S1E). These data implied that a negative correlation between TSC2 and Glrx existed in LAM. It has been reported that loss of TSC2 leads to mTORC1 hyperactivation and further, increased phosphorylation of S6K, S6 and 4E‐BP1.^31^ Thus, to determine if mTORC1 is necessary for TSC2‐mediated Glrx activation, LAM‐derived cells were treated with Rapamycin, a mTORC1 inhibitor. Unexpectedly, Immunoblot and RT‐qPCR assays showed that the expression of Glrx in TSC2‐deficient 621‐101(Figure 2A and Figure S1B), ELT3 (Figure S1D) and 293T cells (Figure S1E) was rarely affected by mTORC1 inhibition. In addition, there was also no significant change of Glrx expression in 621‐101 cells after knockdown *RAPTOR*, a critical component of mTORC1 (Figure 2B, left panel). However, Torin1 treatment, mediating mTORC1/2 inhibition, markedly suppressed Glrx expression in 621‐101 cells (Figure 2C). Similar results were obtained when *RICTOR*, a key component of mTORC2, was knocked down (Figure 2B, right panel), accompanied by higher levels of protein GSH adducts (Figure 2D). Together, these results indicated that TSC2 negatively regulated Glrx activation in a mTORC1‐independent manner, but might relate to mTORC2.

### 3.2 Glrx depletion attenuated cell proliferation and induced apoptosis of TSC2‐deficient cells with exacerbated oxidative stress

**FIGURE 3:**
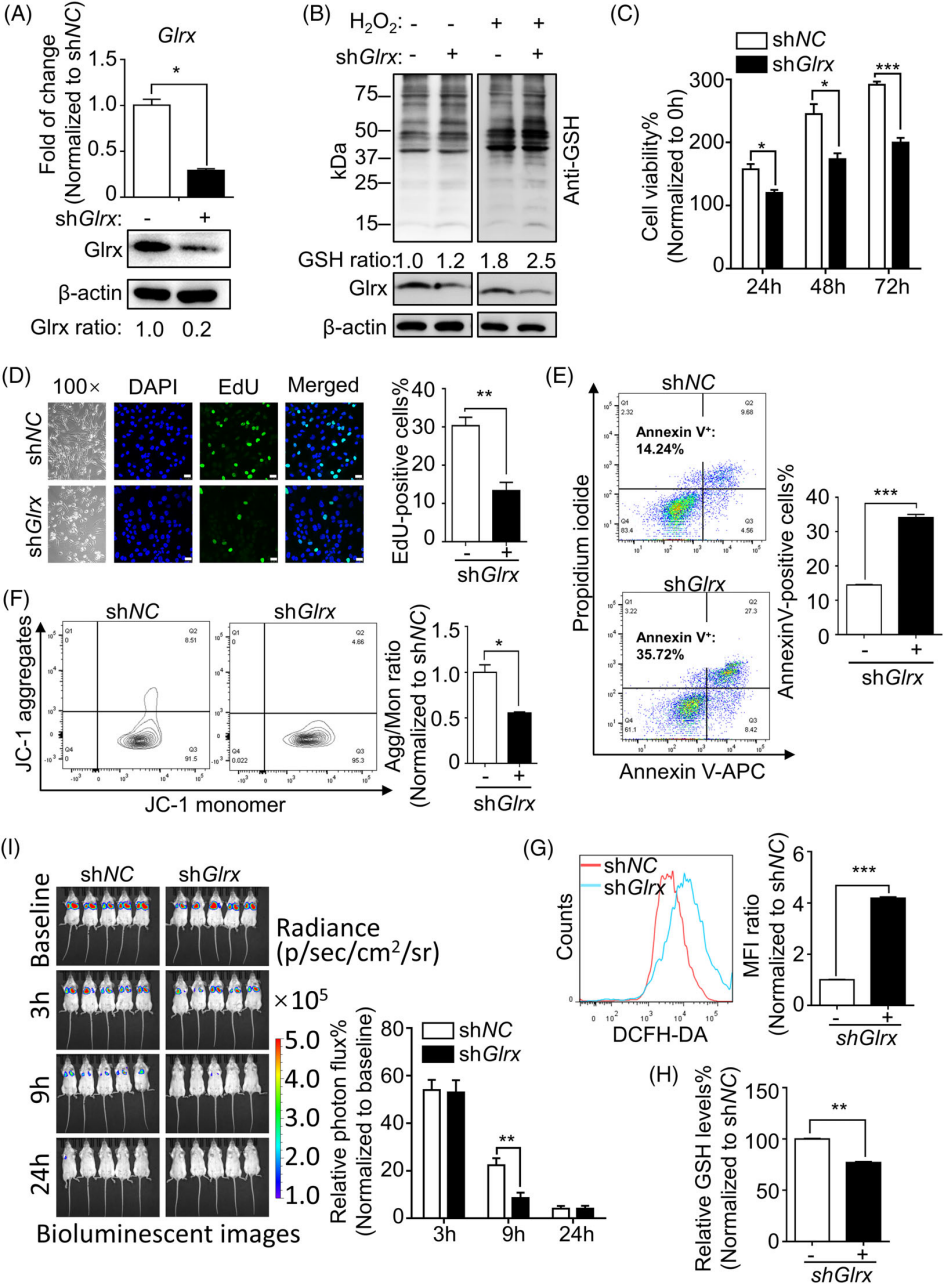
Glutaredoxin‐1 (Glrx) depletion reduced cell survival of TSC2‐deficient 621‐101 cells, related to aberrant oxidative stress and protein glutathione (GSH) adducts. A stable Glrx knockdown 621‐101 cell line was constructed with lentiviral shRNA targeted against Glrx. (A) The knockdown efficiency of Glrx in 621‐101 cells was evaluated by RT‐qPCR and Immunoblot assays, respectively. (B) Immunoblot analysis of protein GSH adducts in Glrx‐depleted 621‐101 cells treated with 300 μM H_2_O_2_. (C) CCK‐8 assay was performed to evaluate cell viability at indicated time in 621‐101 cells after Glrx knockdown. (D) Changes in the morphology of 621‐101 cells and EdU staining images were captured after targeting Glrx. The EdU‐positive cells % were quantified. magnification ×200; Scale Bar, 25 μm. (E) The apoptotic cell numbers (Annexin V positive staining) in 621‐101 cells after transfection with control or *Glrx* shRNA were assessed and quantified by flow cytometry. (F) Flow cytometry analysis of mitochondrial membrane potential (MMP) in 621‐101 cells, labelling with the fluorescent probe JC‐1. Impaired MMP was quantified and indicated by the JC‐1 aggregates/monomer (Agg/Mon) ratio. (G) Intracellular reactive oxygen species (ROS) was determined by flow cytometry. The mean fluorescence intensity (MFI) ratio was quantified to indicate the cellular ROS level. (H) Cellular GSH levels were measured by kit. (I) 621‐101 luciferase‐expressing cells transfected with *Glrx* shRNA or control shRNA were intravenously injected into BALB/c mice (*n* = 5). Bioluminescence intensity was monitored and total photon flux was quantified at 0, 3, 9 and 24 h post injection. Student's *t*‐test, *p < .05, **p < .01, ***p < .001.

To define the role of Glrx on the proliferation of LAM, lentiviral shRNA targeted against *Glrx* was used to construct a stable 621‐101 cell line with Glrx knockdown. The RT‐qPCR and Immunoblot analysis were performed to evaluate the efficiency of *Glrx* knockdown (Figure 3A). Furthermore, knocking down Glrx increased the levels of protein GSH adducts with the stimulation of H_2_O_2_, implying loss of Glrx activity and an elevated sensitivity toward oxidative stress after Glrx depletion in TSC2‐deficient 621‐101 cells (Figure 3B). Subsequently, cell apoptosis and cell cycle assay were performed by flow cytometry analysis. CCK8 assay, colony formation and EdU staining were performed to assess cell viability, colony formation ability and proliferation, respectively. The results suggested that depletion of Glrx reduced cell viability (Figure 3C) and colony formation ability (Figure S2C), inhibited cell proliferation (Figure 3D and Figure S2A), caused cell cycle G1 phase arrest (Figure S2B), and particularly induced apoptosis of 621‐101 cells (Figure 3E). On the contrary, overexpression of Glrx promoted cell survival in 621‐103 cells (Figure S2D).

As an essential thioltransferase, Glrx contributes to the regulation of redox signalling by catalyzing the removal of protein‐bound GSH without exerting direct antioxidant properties.^32^ To determine the role of Glrx in defending oxidative stress in LAM, the level of ROS was measured. Interestingly, we observed that Glrx depletion enhanced significantly accumulation of ROS (Figure 3G), decreased the level of GSH (Figure 3H) and antioxidant superoxide dismutase (SOD) expression, while altered expression of NADPH oxidase 4 (Nox4) and nuclear factor erythroid 2‐related factor 2 (Nrf2) redox factors in 621‐101 cells (Figure S2G). These implied that Glrx depletion exacerbated oxidative stress and might be responsible for cell death in 621‐101 cells. In addition, as the major source of ROS and the major target of ROS damage,^33^ MMP analysis showed that Glrx depletion destroyed mitochondrial membrane permeability with the loss of MMP (Figure 3F). This event also provided an early indication for the initiation of cell apoptosis. Similarly, targeting Glrx triggered apoptosis and decreased MMP in ELT3‐V3 cells (Figures 2E,F).

To further examine the effects of Glrx on LAM cell survival in a preclinical model, 621‐101 luciferase‐expressing cells with control or Glrx‐depletion were injected into BALB/c mice intravenously. Following intraperitoneal injection of D‐luciferin, bioluminescence intensity was measured in the chest regions of all mice at the indicated time (Figure S2H). Importantly, lung colonization of Glrx‐depleted cells was dramatically decreased compared with control cells at 9 h after cell injection (Figure 3I). Collectively, these results suggested that Glrx was required for the maintenance of the intracellular redox balance and cell survival of 621‐101 cells, and depletion of Glrx impaired tumorigenesis both in vitro and in vivo.

### 3.3 The upregulation of Bim triggered apoptosis of TSC2‐deficient cells, caused by Glrx depletion

**FIGURE 4:**
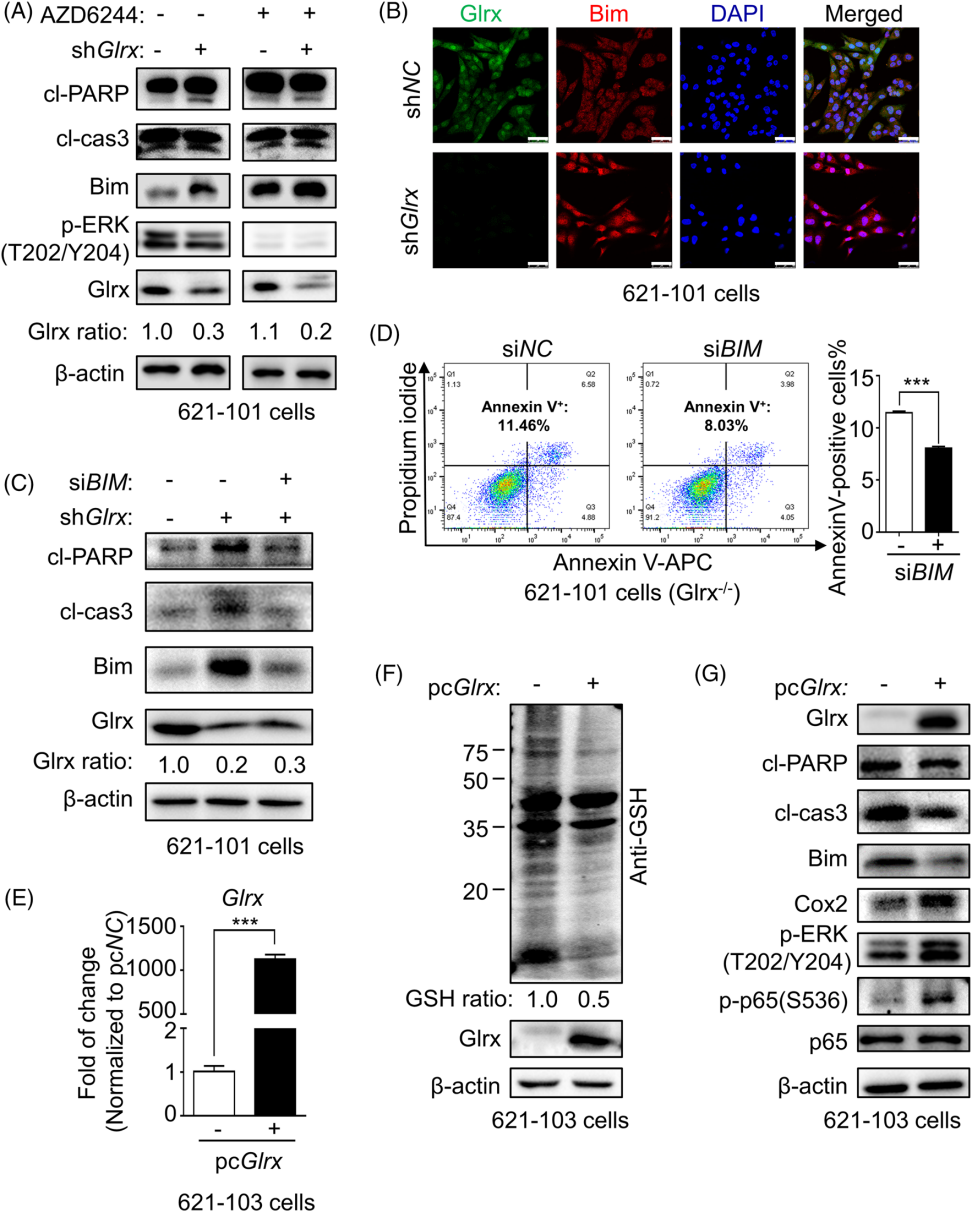
The upregulation of Bim triggered apoptosis of TSC2‐deficient 621‐101 cells, caused by glutaredoxin‐1 (Glrx) depletion. (A) Immunoblot analysis of the levels of Glrx, Bim, cleaved poly‐ADP‐ribose polymerase (cl‐PARP), cl‐caspase3, and p‐ERK1/2(T202/Y204) in 621‐101 cells after knockdown Glrx treated with or without AZD6244. (B) Confocal microscopy images of 621‐101 cells showing Glrx (green) and Bim (red), DAPI was used for nucleus staining. Scale Bar, 50 μm. (C) Immunoblot analysis of the levels of Glrx, Bim, cl‐PARP and cl‐caspase3 in Glrx‐depleted 621‐101 cells transfected with *BIM* siRNA. (D) The apoptotic cell numbers (Annexin V‐positive staining) in Glrx‐deficient 621‐101 cells after transfection with control or *BIM* siRNA were assessed and quantified by flow cytometry. (E) The efficiency of *Glrx* overexpression was measured by RT‐qPCR analysis in 621‐103 cells. (F) Immunoblot analysis of protein glutathione (GSH) adducts confirmed efficient overexpression of Glrx. (G) Immunoblot analysis of the levels of Glrx, Bim, cl‐PARP, cl‐caspase3, Cox2, p‐ERK1/2(T202/Y204), p‐p65(S536) and p65 in 621‐103 cells after Glrx overexpression. Student's *t*‐test, ***p < .001.

Bim (BCL‐2‐interacting mediator of cell death), a proapoptotic member of the Bcl2 family with a BH3‐only domain,^34^ has been reported that the phosphorylation of Bim at serine 69 mediated by ERK1/2 results in its degradation via the proteasome pathway.^35^ Recent studies showed that Glrx protects endothelial cells from oxidative stress‐induced apoptosis by inhibiting Bim.^36^ Moreover, our previous studies have shown that Bim is an essential mediator of anoikis‐type apoptosis in LAM.^37^ Therefore, to further explore the underlying mechanism of Glrx depletion‐induced apoptosis, we performed Immunofluorescence and Immunohistochemistry (IHC) staining of LAM lung tissues. As demonstrated in previous studies,^37^ the level of Bim in alpha‐smooth muscle actin (α‐SMA) or p‐S6(S235/236) positive LAM lesions was lower compared with adjacent lung parenchyma (Figure S3A). Similarly, Immunoblot analysis showed that Bim expression in TSC2‐deficient ELT3‐V3 xenografts was lower compared with TSC2‐addback ELT3‐T3 xenografts (Figure 1E). These results all indicated that Bim might play a critical role in the LAM pathogenesis. Based on these results, we assumed that the protective effect of Glrx on LAM was dependent on Bim degradation. Surprisingly, RT‐qPCR analysis demonstrated that *BIM* mRNA level was not affected by Glrx knockdown (Figure S3B). However, Immunoblot analysis showed that Glrx depletion markedly increased the levels of cleaved poly‐ADP‐ribose polymerase (cl‐PARP) and Bim in 621‐101 cells, accompanied with decreased phosphorylated ERK1/2(T202/Y204) (Figure 4A). Further Immunofluorescence analysis also demonstrated a significant accumulation of Bim in 621‐101 cells after Glrx knockdown (Figure 4B). Furthermore, AZD6244 (MEK1/2 inhibitor) treatment also confirmed that blocking ERK1/2 activation resulted in Bim accumulation in 621‐101 cells (Figure 4A). Next, we overexpressed Glrx in 621‐103 cells. RT‐qPCR analysis of *Glrx* level (Figure 4E) and Immunoblot analysis of protein GSH adducts (Figure 4F) were performed to evaluate the overexpression efficiency and Glrx activity. On the contrary, Glrx overexpression inhibited the expressions of cl‐PARP, cleaved caspase3 (cl‐caspase3) and Bim, together with increased p‐ERK1/2(T202/Y204) in 621‐103 cells (Figure 4G). These results indicated that upregulation of Bim, caused by Glrx depletion might trigger apoptosis of TSC2‐deficient cells.

To further characterize the role of Bim in LAM, Glrx‐depleted 621‐101 cells were transfected with siRNAs targeting *BIM* (mixed si*BIM*#1, si*BIM*#2 and si*BIM*#3) or N.C. *BIM* silencing markedly reduced levels of the cleaved form of PARP and caspase3 (Figure 4C), as well as rescued Glrx depletion‐induced apoptosis in 621‐101 cells (Figure 4D). In addition, three different forms of *BIM* overexpressing plasmids (*BIM, BIM‐SA, BIM‐SD*) were applied to overexpress *BIM* in 621‐101 cells in the presence of Bortezomib (BTZ), the proteasome inhibitor for preventing degradation of Bim. In contrast, BIM‐SA (the constitutively activated type of BIM) overexpression visibly exacerbated levels of cl‐PARP and cl‐caspase3 in Glrx‐depleted and control 621‐101 cells, compared to BIM (the wildtype form) and BIM‐SD (the deficient form) overexpression (Figure S3E). These results indicated that Bim was a critical regulator of Glrx depletion‐promoted apoptosis, and ERK1/2 activation was responsible for Bim proteasomal degradation, which confers LAM‐derived TCS2‐decicient cell survival.

### 3.4 Abnormal Glrx enhanced LAM‐derived TSC2‐deficient cell survival by mediating Cox2/PGE2 biosynthesis

**FIGURE 5:**
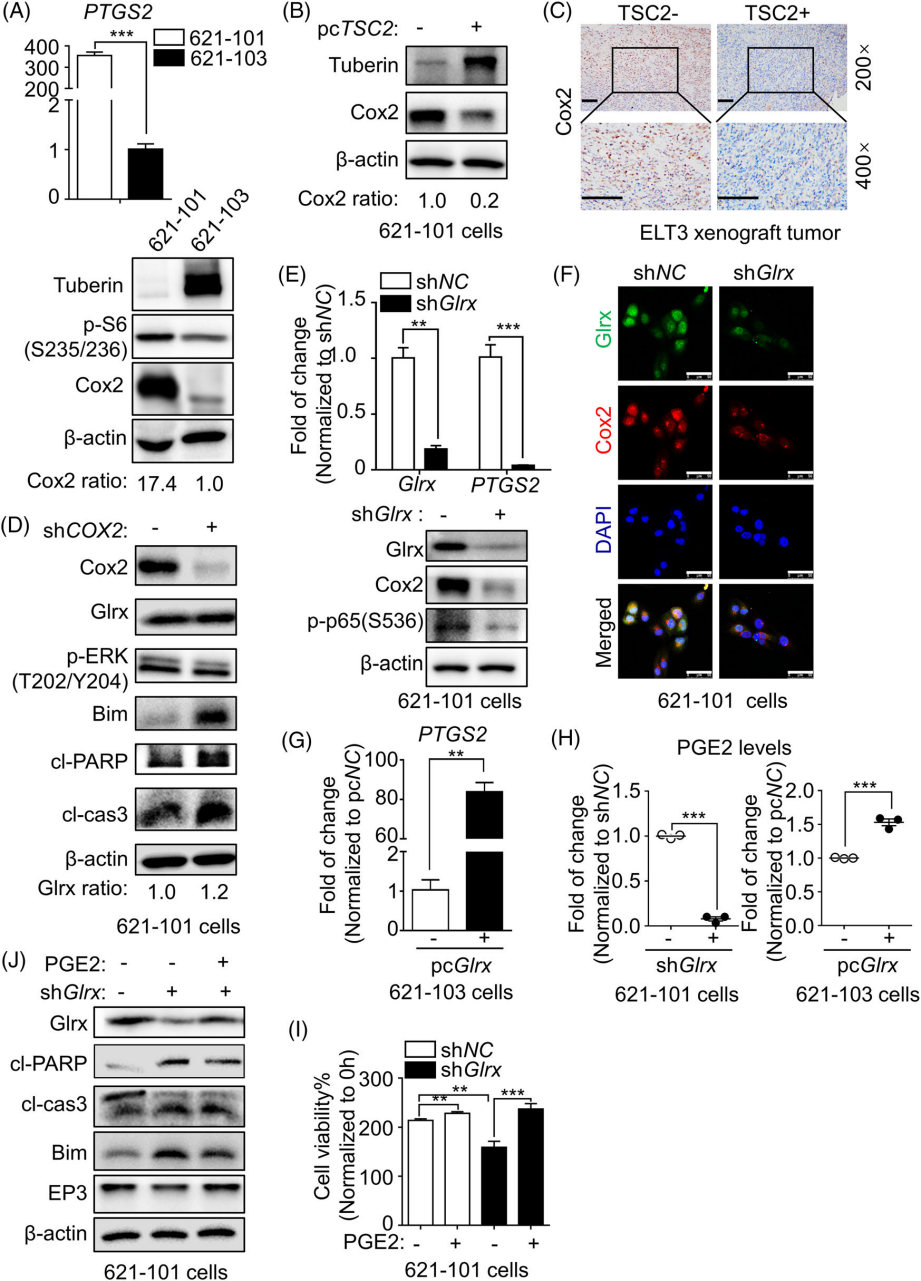
Targeting glutaredoxin‐1 (Glrx) inhibited Cox2‐mediated PGE2 biosynthesis in TSC2‐deficient 621‐101 cells. (A) RT‐qPCR analysis of *PTGS2* and Immunoblot analysis of Tuberin, p‐S6(S235/236), Cox2 in lymphangioleiomyomatosis (LAM)‐derived 621‐101 and 621‐103 cells. (B) 621‐101 cells were transfected transiently with *TSC2*‐overexpressed plasmid for 72 h. Immunoblot analysis of Tuberin and Cox2 expression. (C) Representative Immunohistochemistry (IHC) staining images of Cox2 in ELT3 tumour sections were shown. Scale Bar, 100 μm. (D) Immunoblot was performed for Cox2, Glrx, p‐ERK1/2 (T202/Y204), Bim, cleaved poly‐ADP‐ribose polymerase (cl‐PARP) and cl‐caspase3 in control or *COX2* shRNA transfected 621‐101 cells. (E) RT‐qPCR analysis (upper) of *Glrx* and *PTGS2*, and Immunoblot analysis (lower) of Glrx, Cox2, p‐p65(S536) in 621‐101 cells after knockdown Glrx. (F) Representative images of Glrx and Cox2 in 621‐101 cells after transfection with control or *Glrx* shRNA assessed by Immunofluorescence. DAPI was used for nucleus staining. Scale Bar, 50 μm. (G) RT‐qPCR analysis of *PTGS2* in 621‐103 cells transiently transfected with *Glrx* overexpressed plasmids. (H) The cell culture supernatants were collected and the levels of secreted PGE2 were measured using ELISA kits. (I) Cell viability was measured by CCK8 assay in 621‐101 sh*NC* and sh*Glrx* cells with 5 μM PGE2 treatment for 24 h. (J) Immunoblot was performed for Glrx, Bim, EP3, cl‐PARP and cl‐caspase3 in 621‐101 sh*NC* and sh*Glrx* cells with 5 μM PGE2 treatment. Student's *t*‐test, **p < .01, ***p < .001.

Cyclooxygenase‐2 (Cox2 or PTGS2), an inducible isoform of prostaglandin synthases (PTGS), can transform arachidonic acid to Prostaglandin H2 (PGH2), a precursor for Prostaglandins.^38^ Prostaglandin E2 (PGE2), an efficient pro‐inflammatory mediator, is involved in diverse cellular processes via four G protein‐coupled receptors, termed EP receptors 1−4.^39^ Recent studies highlighted that the activation of Raf‐MEK‐ERK1/2 pathway, mediated by Cox2/PGE2, acts as a crucial negative regulator of Bim expression in colorectal tumour.^40^ Accordingly, to further investigate the underlying mechanism of Glrx regulated Bim expression, Immunoblot and RT‐qPCR analysis were performed to examine Cox2 expression in LAM‐derived cells. Consistent with our previous results,^41^, ^42^ higher level of Cox2 was detected in 621‐101 cells compared to 621‐103 cells (Figure 5A). Similar results were found in ELT3 cells (Figure S4A) and xenograft tumors of ELT3 cells (Figures 1E and 5C). Meanwhile, the negative relationship between TSC2 and Cox2 was further verified via overexpression of TSC2 in 621‐101 cells (Figure 5B).

To further determine the mechanism of ERK1/2 inactivation after Glrx knockdown in LAM, Cox2 expression was measured after Glrx knockdown in 621‐101 cells. Excitingly, Glrx depletion in 621‐101 cells obviously decreased Cox2 expression (Figure 5E,F), together with lower level of PGE2 in cell culture supernatants by ELISA assay (Figure 5H, left panel). On the contrary, Glrx overexpression increased Cox2 expression (Figures 4G and 5G) and PGE2 secretion (Figure 5H, right panel) in 621‐103 cells. Meanwhile, it was worth noting that Cox2 knockdown also promoted the expressions of cl‐PARP, cl‐caspase3 and Bim, together with decreased p‐ERK1/2(T202/Y204), while the expression of Glrx was rarely affected in 621‐101 cells (Figure 5D). As described in 621‐101 cells, similar alterations of cl‐PARP, cl‐caspase3, Bim, Cox2 and p‐ERK1/2(T202/Y204) were observed after targeting Glrx in ELT3 cells (Figures 3D and 4B), accompanied with increased protein GSH adducts (Figure S3C). These findings supported that Cox2 acted as a downstream target of Glrx to mediate PGE2 biosynthesis, which might further contribute to ERK1/2 activation and Bim degradation. To elucidate the hypothesis, exogenous PGE2 was added after Glrx depletion in 621‐101 cells. We noticed that PGE2 supplement rescued cell viability (Figure 5I), reversed the accumulation of Bim and the reduction of EP3 (Figure 5J). In summary, these findings suggested that Glrx could enhance 621‐101 cells survival by mediating Cox2‐PGE2 biosynthesis, which further promoted ERK1/2 phosphorylation and Bim degradation.

### 3.5 Glrx promoted Cox2 expression via modulating NF‐κB p65 signalling in LAM

**FIGURE 6:**
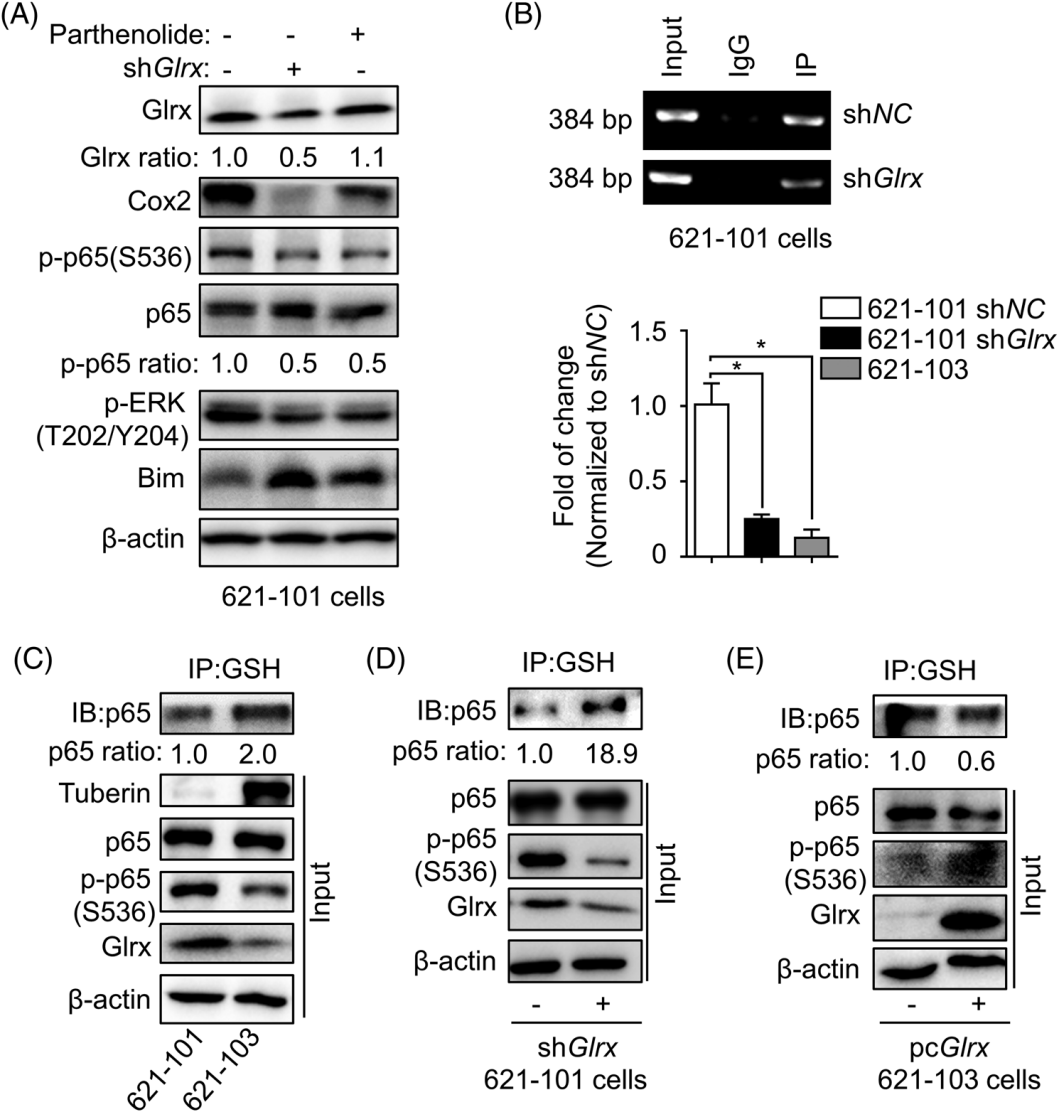
Glutaredoxin‐1 (Glrx) promoted Cox2 expression via modulating nuclear factor‐kappa B (NF‐κB) p65 signalling in lymphangioleiomyomatosis (LAM). (A) 621‐101 cells were treated with 25 μM Parthenolide. the levels of Glrx, Cox2, p‐p65(S536), p65 and p‐ERK1/2(T202/Y204) and Bim were analyzed by Immunoblot assay. (B) Chromatin immunoprecipitation (ChIP) PCR was performed to determine gene abundance of Cox2 promoter region in the groups of positive control (Input), negative control (IgG) and immunoprecipitated (IP), which were Immunoprecipitated with anti‐p‐P65 (S536) antibody in 621‐101 sh*NC* and sh*Glrx* cells (upper). And gene abundance of the Cox2 promoter region was further determined in LAM‐derived cells and Glrx‐depleted 621‐101 cells by RT‐qPCR analysis. (C–E) The extracts from 621‐101 and 621‐103 cells, 621‐101 sh*NC* and sh*Glrx* cells, and 621‐103 cells transfected with control or Glrx overexpressed plasmids were prepared for Immunoprecipitation using anti‐glutathione (GSH) antibody and then the levels of p65 GSH adducts were evaluated by Immunoblot using anti‐p65 antibody. Student's *t*‐test, *p < .05.

The NF‐κB pathway plays a central role in mediating Cox2 expression and can be dynamically regulated through reversible cysteine oxidations of the family members.^43^, ^44^ Recent studies have shown that Glrx activates NF‐κB signalling by attenuating *S*‐glutathionylation of p65 in neurodegenerative diseases.^25^ To clarify the precise mechanisms of regulating Cox2 expression by Glrx in LAM, the expression of phospho‐NF‐κB p65 (p‐p65) on serine residue 536 was detected by Immunoblot and we found that Glrx depletion downregulated p‐p65(S536) expression in 621‐101 cells (Figure 6A). Conversely, Glrx overexpression remarkably elevated the phosphorylation of p65 in 621‐103 cells (Figure 4G). Furthermore, pharmacological inhibition of NF‐κB by parthenolide also diminished the expressions of Cox2, p‐p65(S536), p‐ERK1/2(T202/Y204) and promoted the expression of Bim in 621‐101 cells (Figure 6A). These results implied that p65 played a key role in Cox2 expression regulated by Glrx.

Next, the potential role of p65 was further studied. We queried the promoter sequence of Cox2 to detect whether the p65 signal promotes Cox2 expression by directly binding to the promoter of Cox2. Potential binding sites for p65 (TGGCGTTTCC) were discovered in the promoter region of Cox2 (Figure S4E). And the results demonstrated that p‐p65 could bind to the promoter region of Cox2 (Figure 6B, upper panel). Moreover, RT‐qPCR analysis of ChIP products further indicated that p‐p65 and Cox2 promoter regions have less binding in Glrx‐depleted 621‐101 and 621‐103 cells with lower Cox2 levels (Figure 6B, lower panel).

Furthermore, considering the role of Glrx in controlling GSH adducts, whether the regulation of Cox2 by Glrx is achieved through modulating p65 GSH adducts. Protein GSH adducts were immunoprecipitated (IP) using anti‐GSH antibody and the level of p65 was valued in the IP product by Immunoblot. Increased p65 GSH adducts were observed following Glrx depletion in 621‐101 cells (Figure 6D), which might attenuate the phosphorylation of p65. Besides, the correlation of Glrx and p65 was also confirmed in LAM‐derived cells (Figure 6C) or 621‐103 cells transfected with plasmid overexpressing *Glrx* (Figure 6E). These results demonstrated that Glrx might modulate the NF‐κB pathway and Cox2 expression via balancing p65 phosphorylation and protein GSH adducts production in LAM.

### 3.6 Glrx depletion suppressed tumour growth and induced apoptosis in 621‐101 xenograft tumors

**FIGURE 7:**
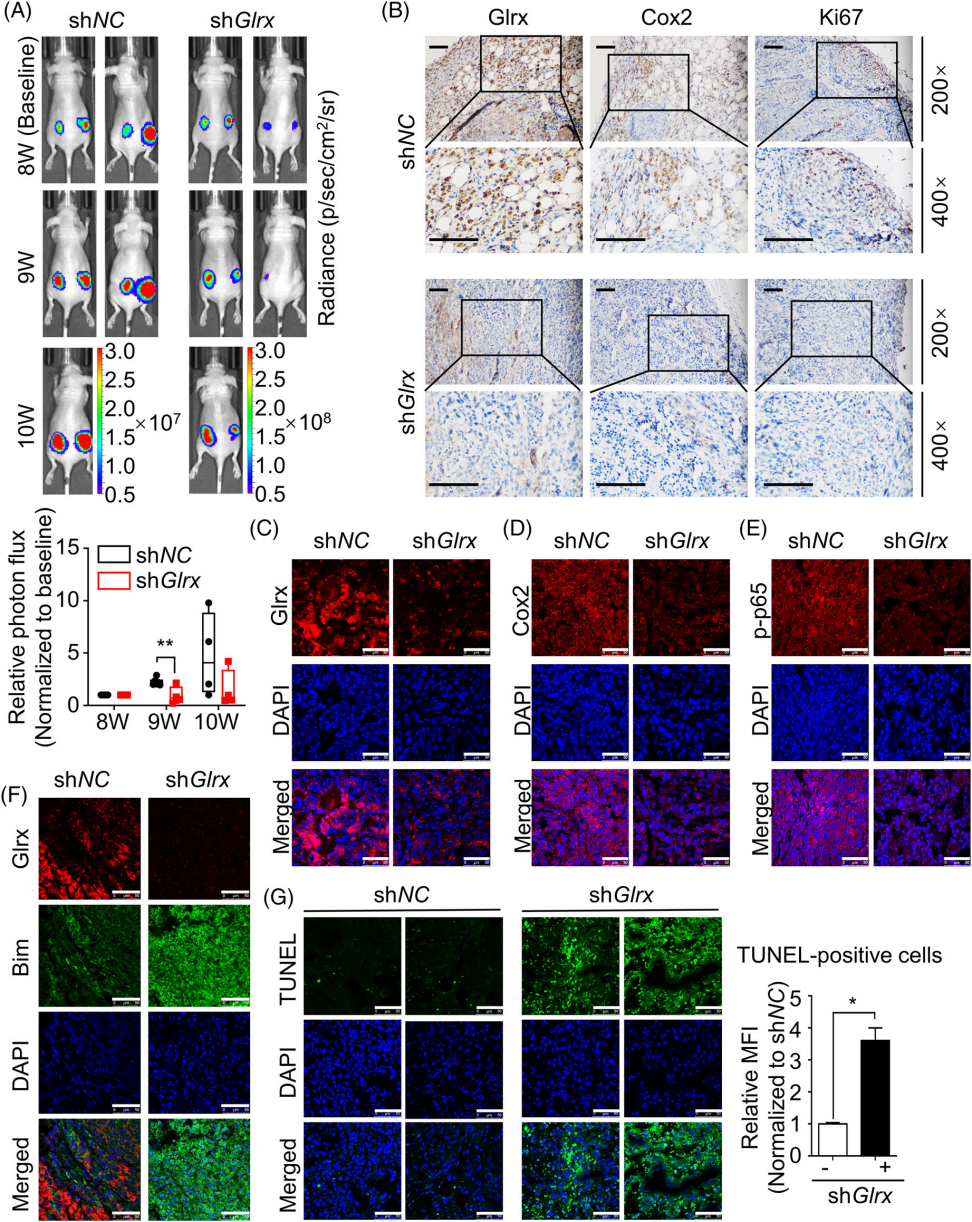
Glutaredoxin‐1 (Glrx) depletion suppressed tumour growth and induced apoptosis in xenograft tumours of 621‐101. (A) Nude mice were inoculated subcutaneously with 621‐101 luciferase‐expressing cells after transfection with control or *Glrx* shRNA. Bioluminescence intensity was monitored and relative photon flux was quantified at the indicated times. (B) Immunohistochemistry (IHC) staining images of Glrx, Cox2 and Ki67 were shown. Scale Bar, 100 μm. (C–E) Representative images of Glrx, Cox2, p‐p65(S536) in xenograft tumours were shown by Immunofluorescence analysis. Scale Bar, 50 μm. (F) The expression and distribution of Glrx and Bim in xenograft tumors were determined by Immunofluorescence assay. Scale Bar, 50 μm (G) TUNEL staining of apoptotic cells was shown and quantified. Scale Bar, 50 μm. Student's *t*‐test, *p < .05, **p < .01.

To further assess the effects of Glrx in vivo, BALB/c females were injected subcutaneously in both flanks with luciferase‐expressing 621‐101 sh*NC* or sh*Glrx* cells. Bioluminescence intensity and tumour volume were monitored once palpable tumour nodules appeared. After 11 weeks, tumors were removed and representative images of tumors in nude mice were recorded (Figure S5A). Compared with sh*NC* group, we observed that Glrx depletion significantly diminished the tumour volume growth ratio (Figure S5B) and displayed a lower growth rate of bioluminescence intensity (Figure 7A). IHC staining analysis showed the reduced levels of Glrx and Cox2, also with a decreased expression of Ki67 in tumors derived from Glrx knockdown cells (Figure 7B). Targeting Glrx showed decreased expressions of Glrx, Cox2, p‐p65(S536) in xenograft tumors assessed by Immunofluorescence staining (Figure 7C–E). Furthermore, Immunoblot analysis showed higher levels of Bim and cl‐PARP and a lower p‐ERK expression in sh*Glrx* tumour tissues (Figure S5C). Similarly, Immunofluorescence staining showed markedly increased Bim level in sh*Glrx* group, as well as lower Bim expression in Glrx‐positive tissue areas compared with adjacent areas (Figure 7F), which was consistent with studies in vitro and human lung lesions. Finally, TUNEL assay of xenograft tumour sections showed more apoptotic cells in the sh*Glrx* group (Figure 7G), indicating that Glrx depletion induced apoptosis in vivo and as a result, led to tumour suppression. Overall, these results indicated that Glrx exacerbated tumorigenesis by enhancing phosphorylation of p65, promoted Cox2 expression and Bim degradation, which attenuated cell apoptosis in vivo.

## 4 DISCUSSION

LAM, a typical representation of rare diseases, is an incurable multisystem disease.^45^ Currently, therapeutic agent of LAM focus on mTOR inhibitors and the efficacy of them has been proved. As the pathogenesis of LAM is being further clarified, other therapeutic agents such as VEGFD inhibitors, statins, chloroquine analogs, cyclin‐dependent kinase inhibitors, matrix metalloproteinase inhibitors, and their combinations also are used in LAM. However, all of them are not curable and the lung transplantation is only chance for LAM patients. Therefore, it is urgent to discover new therapeutic targets. Protein cysteines can undergo various post‐translational modifications that have profound effects on their function and properties. Especially, Thiol groups on protein cysteines are typically considered sensitive to oxidative modifications.^46^ With abundant GSH levels in cells, protein GSH adducts have been implicated as a fundamental mechanism of redox signaling and modulate various cellular activities, such as metabolism, angiogenesis, gene transcription and apoptosis.^9^ Generally, cells generate oxidants and initially oxidize protein thiolates (R‐S^−^) to form sulfenic acid (R‐SOH) or *S*‐nitrosothiols (R‐SNO). They can be reversibly reduced by reacting with GSH and further deglutathionylation. In the process, Glrx, acting as an essential GSH‐dependent thioltransferase, modulates redox signaling by mainly catalyzing deglutathionylation of protein GSH adducts. As a consequence, protein GSH adducts and Glrx contribute to maintain the redox homeostasis under physiological conditions. However, once excessive generation of oxidative stress, the protein cysteine residues are modified and form irreversible sulfonic acid (R‐SO_3_H), which can induce permanent changes in protein structure and function.^9^ Over the last several years, protein GSH adducts have been recognized on various proteins and complex redox regulation by protein GSH adducts and Glrx has been reported involved in various diseases, such as pulmonary fibrosis,^23^ non‐alcoholic fatty liver disease,^32^ neurodegenerative^47^ and cardiovascular diseases.^48^ In addition, recent evidence also showed the reduced Glrx expression and elevated accumulation of PSSG in activated hepatic stellate cells (HSCs). And pirfenidone treatment significantly inhibits liver fibrosis by inducing Glrx expression in HSCs by a Stat5‐dependent manner, which strongly addressed the key role of Glrx in liver fibrosis.^49^ However, redox regulation via protein GSH adducts and Glrx remains poorly understood in LAM.

Here, we showed abnormal activation of Glrx in TSC2‐deficient 621‐101 cells. Remarkably, TSC2 negatively regulated Glrx hyperactivation in a Rapamycin‐insensitive manner. Additionally, to determine the effects of Glrx in the progression of LAM, we knocked down Glrx in 621‐101 cells and the activity of Glrx was evaluated by the content of protein GSH adducts. Our results showed that Glrx depletion in 621‐101 cells significantly inhibited cell proliferation and triggered cell apoptosis, which was mediated by the accumulation of ROS, together with elevated protein GSH adducts. Moreover, the increased Bim expression also contributed to the induction of apoptotic cell death. In preclinical models, we also revealed that Glrx knockdown reduced lung colonization of TSC2‐deficient cells and suppressed tumour growth, supporting that Glrx was a critical regulator of the survival of TSC2‐deficient cells in vivo.

Bim is subjected to ERK1/2‐catalysed serine phosphorylation and further promotes proteasome‐dependent degradation.^50^ Increasing evidences demonstrate the loss of Bim promotes the occurrence of a variety of cancers, including prostate cancer, and endometrial cancer, suggesting a tumour‐suppressive role in these malignancies.^51^, ^52^ In our studies, we found that there was no change at the transcriptional level of Bim, but abnormal accumulation of Bim was observed by Immunoblot analysis after Glrx depletion in 621‐101 cells. Further studies suggested that decreased phosphorylation of ERK1/2 was responsible for Bim accumulation in Glrx‐depleted 621‐101cells.

**FIGURE 8:**
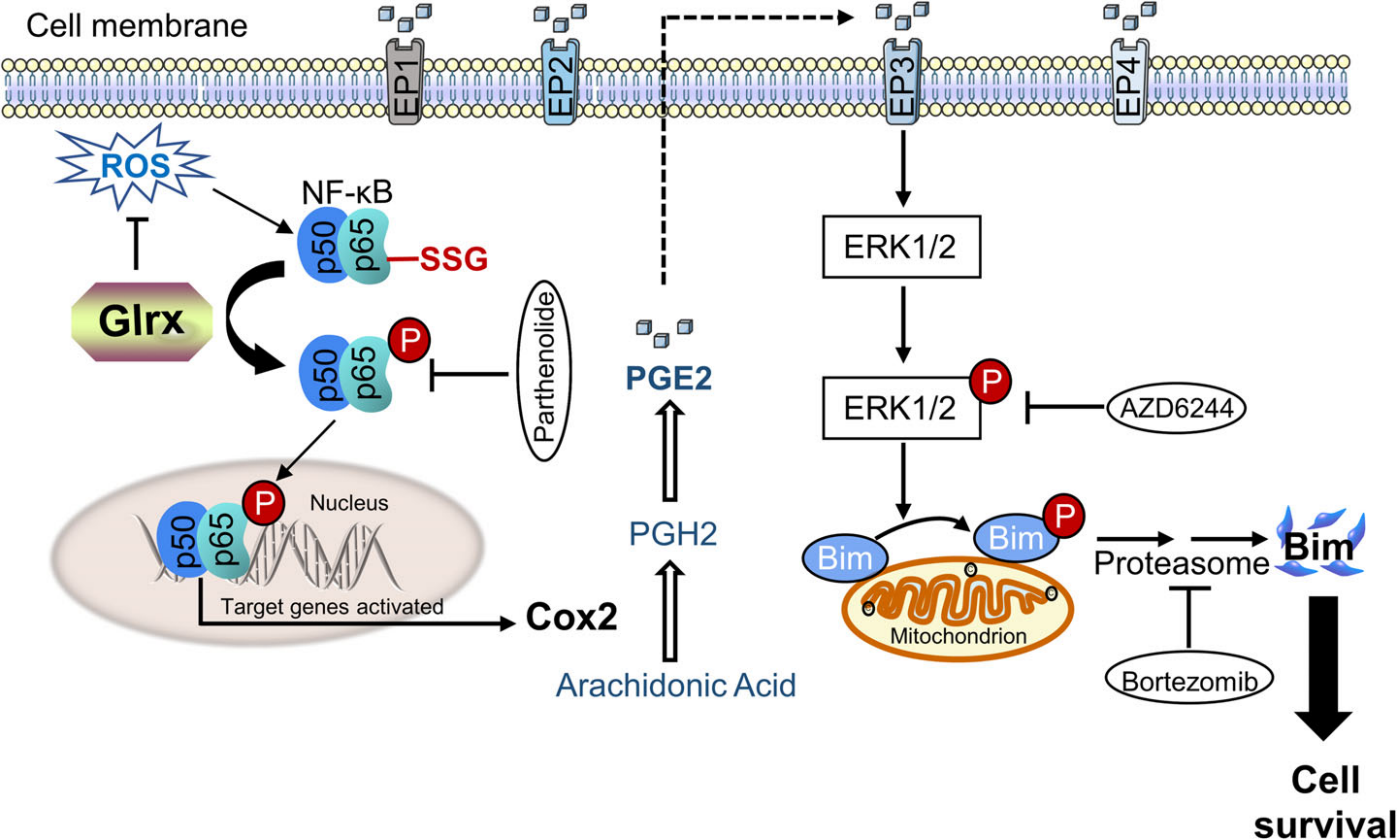
Hypothetical scheme of glutaredoxin‐1 (Glrx) promoted cell survival in lymphangioleiomyomatosis (LAM). (i) The decreased reactive oxygen species (ROS) level; (ii) Promoted Cox2 expression and PGE2 biosynthesis; (iii) The increased phosphorylation of ERK1/2 enhanced phosphorylation of Bim and degradation by the proteasome, which attenuated cell apoptosis.

Next, we further determined the mechanism of ERK1/2 inactivation caused by Glrx knockdown in LAM. Cyclooxygenase‐2 (Cox2), a rate‐limiting enzyme of PGE2 biosynthesis, has been proven to overexpression in 621‐101 cells and showed beneficial anti‐tumour effects by aspirin treatment.^41^ In addition, recent in vitro and preclinical evidence also showed that celecoxib treatment, a Cox2 specific inhibitor, resulted in a 50% decrease in renal cystadenomas volume of Tsc2^+/−^ mice. And a phase I safety study (COLA; NCT02484664) has established the safety of celecoxib with 200 mg orally daily in LAM patients. These results indicated that Cox2 inhibition may provide clinical benefit in LAM patients with mild disease.^53^ However, how TSC2 regulates Cox2 expression is still unclear. The NF‐κB family mediated Cox2 expression and was involved in cancer progression and resistance to treatment.^54^ The NF‐κB pathway is activated by proteasomal degradation of IκBα, leading to NF‐κB release and translocation to nuclear, where it binds to DNA to modulate target gene expression, like Cox2, IL‐1β and IL‐6.^55^ It has been reported that protein GSH adducts at multiple points inhibits the NF‐κB pathway, including p50, p65 and IKK‐β subunits.^56^ Our studies showed that Glrx promoted Cox2 expression and PGE2 biosynthesis, due to decreased p65 GSH adducts formation and increased phosphorylation of p65 in LAM. Furthermore, exacerbated PGE2 metabolism facilitated phosphorylation of ERK1/2, which led to Bim phosphorylation and proteasomal degradation, thus contributing to the progress of LAM (Figure 8). Here, consistent expressions of IL‐1β and IL‐6 with Cox2 also indirectly confirmed the p65 inhibition regulated by Glrx depletion (Figure S4C,D). Furthermore, according to recent reports, LAM patient plasma showed increased IL‐6 relative to healthy controls, and IL‐6 blockade inhibited the proliferation and migration of TSC2‐deficient cells.^57^ These findings indicated that differently from the administration of Cox2 inhibitors for the treatment of LAM, targeting Glrx might exert better anti‐tumour effects through multiple pathways rather than only regulating Cox2 expression.

In conclusion, we demonstrate that TSC2 negatively regulates Glrx overactivation in a mTORC1‐independent manner and the essential role of Glrx on tumorigenesis in LAM. At the same time, the underlying mechanism of Glrx depletion‐induced apoptosis is discussed in TSC2‐deficient 621‐101 cells. As discussed above, these results indicate that Glrx may be an attractive potential therapeutic strategy in the treatment of LAM or other TSC‐related diseases.

## CONFLICT OF INTEREST STATEMENT

The authors declare no conflict of interest.

## Supporting information



Supporting Information

## Data Availability

All data generated or analyzed during this study are included in this published article and are available from the corresponding author on a reasonable request.
