# Supplementary material for: Glutaredoxin‐1 promotes lymphangioleiomyomatosis progression through inhibiting Bim‐mediated apoptosis via COX2/PGE2/ERK pathway
Source: Clin Transl Med. 2023 Jul 21;13(7):e1333. doi: 10.1002/ctm2.1333 (PMC10361546; doi:10.1002/ctm2.1333)
Supplement: Supplementary file 1 — Supporting Information [file CTM2-13-e1333-s001.docx]

Supplementary Materials for

**Glrx promotes LAM progression through inhibiting Bim-mediated apoptosis via**

**COX2/PGE2/ERK pathway**

**The file includes:**

Supplementary methods

Supplementary tables

Supplementary figure 1 to 5

Legends for Supplementary figure 1 to 5

**Supplementary methods**

**Gene Expression Profiling Interactive Analysis (GEPIA)**

The online database GEPIA (http://gepia.cancer-pku.cn/index.html) was used to analyze the differential expression of Glrx in different tumors and normal tissue datasets. GEPIA is a novel interactive web server to analyze the RNA sequencing data based on TCGA and the GTEx projects. GEPIA also provides the option of conducting Overall survival (OS) or disease-free survival (DFS) analysis based on relative gene expression levels by the log-rank test and Mantel-Cox test. The correlation between *TSC2* and *Glrx* mRNA expression was evaluated using the GEPIA database.

**Supplementary table 1.** **List of primers used for RT-qPCR**

| **Primer name** | **Primer sequences (5'to3')** |
| --- | --- |
| human β-actin forward | CACCATTGGCAATGAGCGGTTC |
| human β-actin reverse | AGGTCTTTGCGGATGTCCACGT |
| human TSC2 forward | CCAAACCAACAAGCAAAGATTCA |
| human TSC2 reverse | CACATTCCATGCTCAGTTCTCT |
| human Glrx forward | CCCATCAAACAAGGGCTTCTG |
| human Glrx reverse | CTGCATCCGCCTATACAATCTT |
| human PTGS2 forward | CTGGCGCTCAGCCATACAG |
| human PTGS2 reverse | CGCACTTATACTGGTCAAATCCC |
| human BIM forward | TAAGTTCTGAGTGTGACCGAGA |
| human BIM reverse | GCTCTGTCTGTAGGGAGGTAGG |
| human HMOX1 forward | AAGACTGCGTTCCTGCTCAAC |
| human HMOX1 reverse | AAAGCCCTACAGCAACTGTCG |
| human NFE2L2 forward | TCCAGTCAGAAACCAGTGGAT |
| human NFE2L2 reverse | GAATGTCTGCGCCAAAAGCTG |
| human SOD forward | GGTGGGCCAAAGGATGAAGAG |
| human SOD reverse | CCACAAGCCAAACGACTTCC |
| human NOX1 forward | TTGTTTGGTTAGGGCTGAATGT |
| human NOX1 reverse | GCCAATGTTGACCCAAGGATTTT |
| human NOX2 forward | AACGAATTGTACGTGGGCAGA |
| human NOX2 reverse | GAGGGTTTCCAGCAAACTGAG |
| human NOX3 forward | CGTGGCGCATTTCTTCAACC |
| human NOX3 reverse | GCTCTCGTTAGGGGTGTTGC |
| human NOX4 forward | TGTGCCGAACACTCTTGGC |
| human NOX4 reverse | ACATGCACGCCTGAGAAAATA |
| human IL-6 forward | ACTCACCTCTTCAGAACGAATTG |
| human IL-6 reverse | CCATCTTTGGAAGGTTCAGGTTG |
| human IL-1β forward | ATGATGGCTTATTACAGTGGCAA |
| human IL-1β reverse | GTCGGAGATTCGTAGCTGGA |
| rat GAPDH forward | AGACAGCCGCATCTTCTTGTGC |
| rat GAPDH reverse | CTCCTGGAAGATGGTGATGG |
| rat Glrx forward | CACAGGTTCCTCGGGTCTTC |
| rat Glrx reverse | GTTTTTGTCCCCAGCCTCAC |


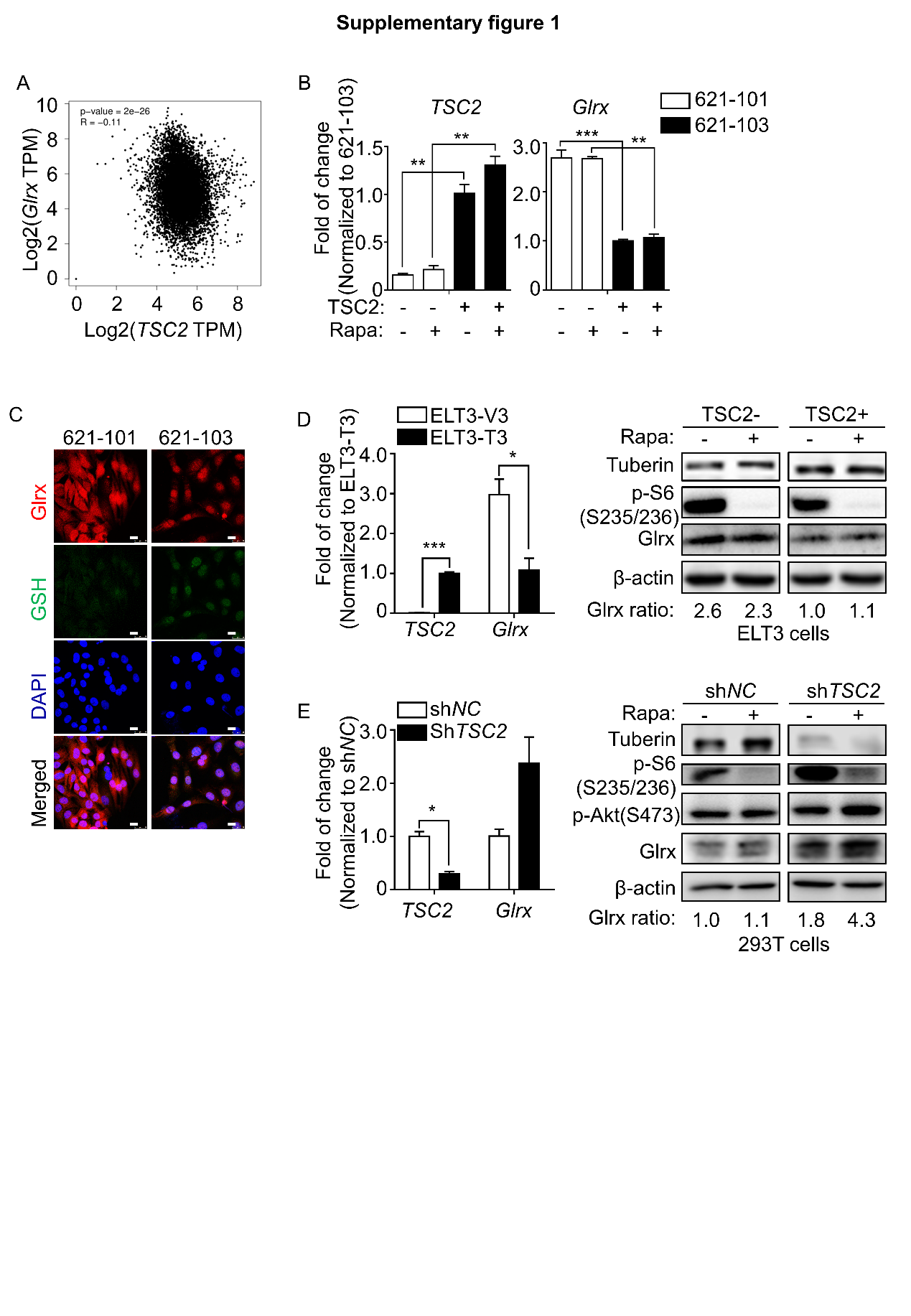


**Supplementary figure 1. TSC2 negatively regulated Glrx in a mTORC1-indepedent manner.** (**A**) GEPIA correlation analysis between *TSC2* and *Glrx* in different types of cancers. (**B**) RT-qPCR analysis of *TSC2* and *Glrx* in LAM-derived 621-101 and 621-103 cells treated with 20 nM rapamycin for 24 h. **(C)** Representative images of protein GSH adducts were detected by Immunofluorescence in LAM-derived 621-101 and 621-103 cells. DAPI was used for nucleus staining. Scale Bar, 25 µm. (**D**) RT-qPCR analysis (left panel) of *TSC2* and *Glrx*, and Immunoblot analysis (right panel) of Tuberin, p-S6(S235/236), Glrx in ELT3-V3 and ELT3-T3 cells. (**E**) RT-qPCR analysis (left panel) of *TSC2* and *Glrx*, and Immunoblot analysis (right panel) of Tuberin, p-S6(S235/236), p-AKT(S473), Glrx in 293T cells transfected with control or *TSC2* shRNA. Student's t-test, *P < 0.05, **P < 0.01, ***P < 0.001.


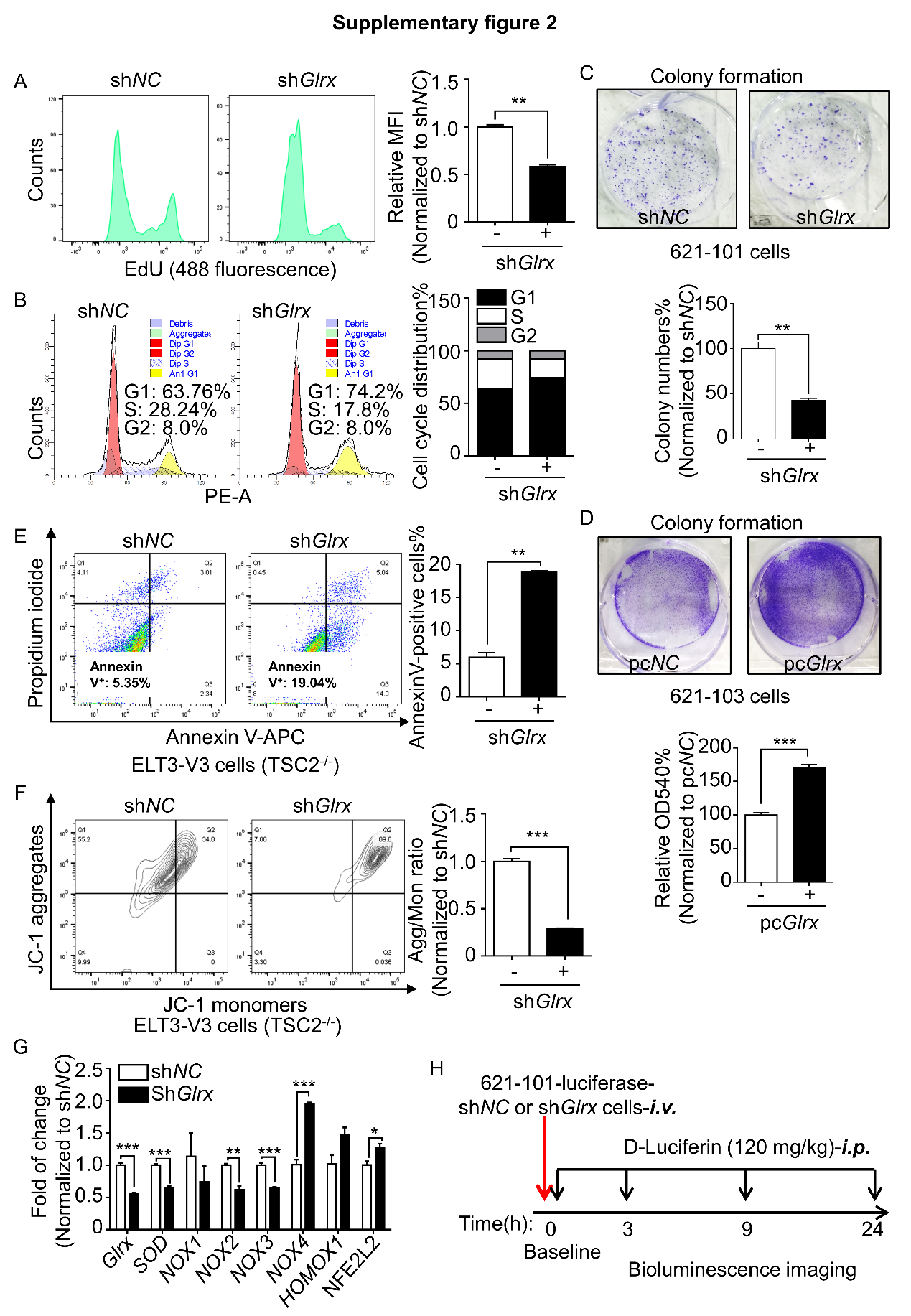


**Supplementary figure 2. Glrx depletion reduced proliferation and induced apoptosis of TSC2-deficient cells.** 621-101 cells were transfected with control or *Glrx* shRNA. (**A**) EdU staining was measured by flow cytometry, and indicated by relative mean fluorescence intensity (MFI). (**B**) Representative flow cytometric data showing the cell cycle distribution and percentages of cells in each phase of the cell cycle. (**C**) Representative images of the colony formation assay and quantification of colony numbers. (**D**) 621-103 cells were transiently transfected with *Glrx* overexpressed plasmids for 3 days and then cells were stained with crystal violet. ELT3-V3 cells were transfected with control or *Glrx* shRNA. (**E**) Apoptosis was measured by flow cytometry, labeling with Annexin V and propidium iodide in ELT3-V3 cells. Cell apoptosis was quantified by Annexin V-positive staining. **(F)** Flow cytometry analysis of mitochondrial membrane potential (MMP) in ELT3-V3 cells, labeling with the fluorescent probe JC-1. Impaired MMP was quantified and indicated by the JC-1 aggregates/monomer (Agg/Mon) ratio. (**G**) RT-qPCR analysis were performed to analyze *Glrx*, *SOD*, *NOX1-4*, *HOMOX1* and *NFE2L2* gene expression in 621-101 cells after Glrx knockdown. (**H**) Schematic protocol for bioluminescent imaging. Student's t-test, **P < 0.05, **P < 0.01, ***P < 0.001.


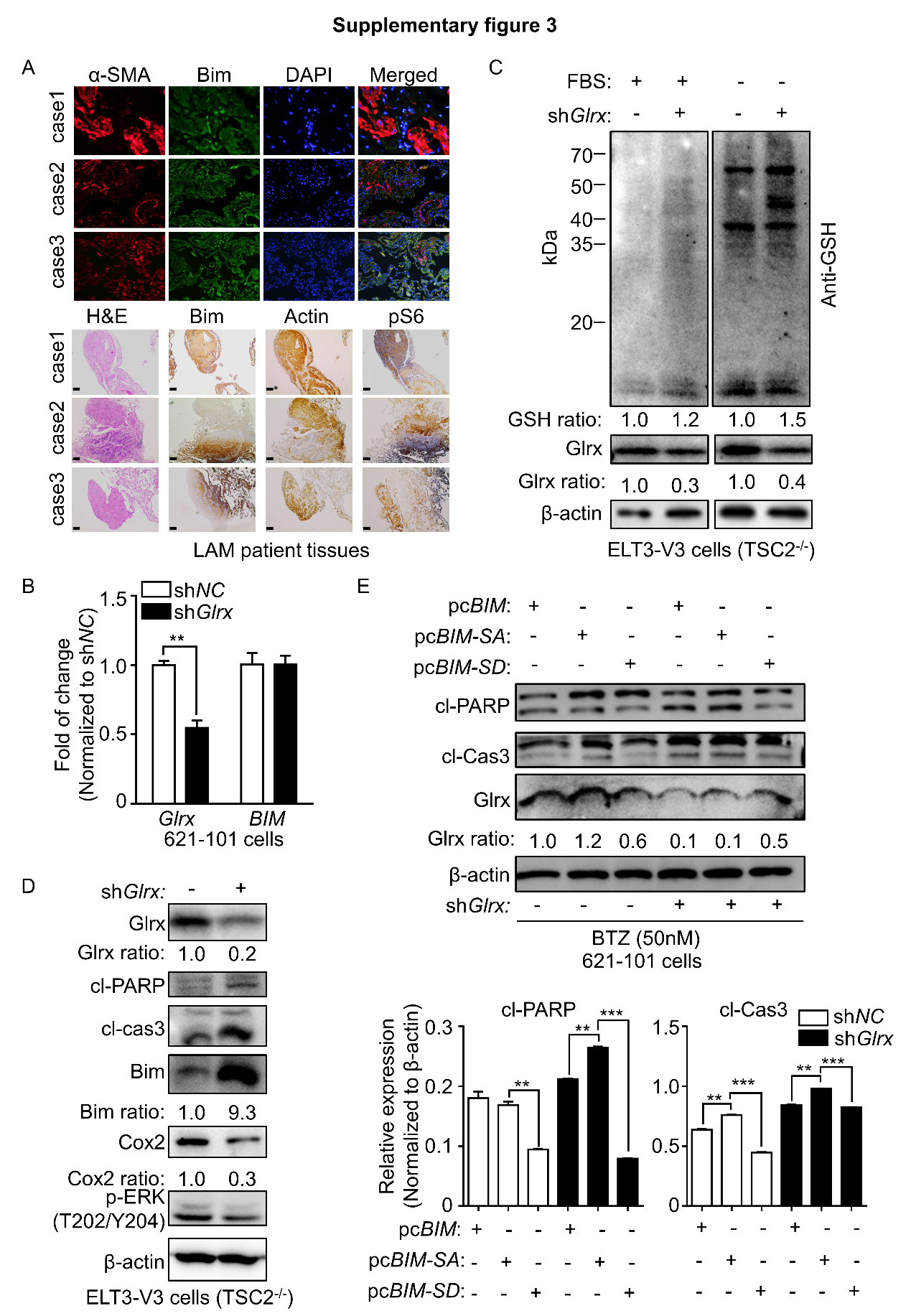


**Supplementary figure 3. The upregulation of Bim triggered cell apoptosis.** (**A**) Immunofluorescence and IHC staining showing the level of Bim in α-SMA or p-S6(S235/236)-positive lung lesions from LAM patients. (**B**) The gene expression of *BIM* was analyzed by RT-qPCR in 621-101 cells after Glrx knockdown. (**C**) Immunoblot analysis of protein GSH adducts in *Glrx*-depleted ELT3-V3 cells treated with or without serum-free DMEM medium. (**D**) Immunoblot analysis of the levels of Glrx, cl-PARP, cl-caspase3, Bim, Cox2, p-ERK1/2(T202/Y204) in ELT3-V3 cells after Glrx depletion. (**E**) 621-101 sh*NC* and sh*Glrx* cells were transiently transfected three different forms of *BIM* overexpressing plasmids with 50 nM Bortezomib treatment, respectively (*BIM*: wild type, *BIM-SA*: the constitutively activated type, *BIM-SD*: inactivated type). Immunoblot analysis of the levels of cl-PARP, cl-caspase3 and Glrx in 621-101 cells.


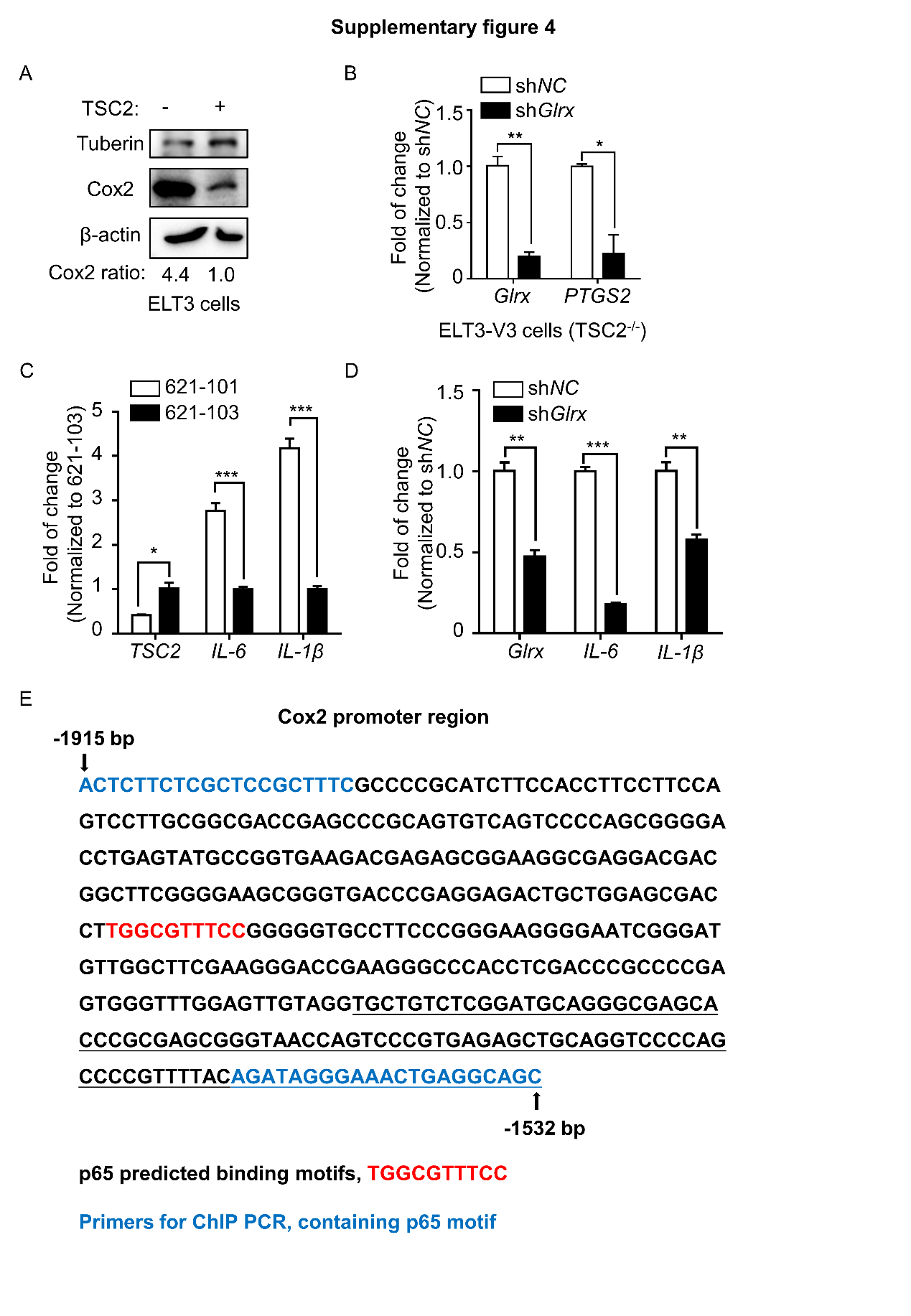


**Supplementary figure 4. Glrx regulated Cox2 expression via balancing P65 signaling in LAM. (A)** The expressions of Tuberin and Cox2 were assessed by immunoblot in ELT3-derived cells. **(B)** The mRNA levels of *Glrx* and *PTGS2* were analyzed by RT-qPCR in ELT3-V3 cells after Glrx depletion. **(C)** The gene expressions of *TSC2* and *IL-6, IL-1β* were assessed by RT-qPCR in LAM-derived 621-101 and 621-103 cells. **(D)** The gene expressions of *TSC2* and *IL-6, IL-1β* were assessed by RT-qPCR in 621-101 cells after Glrx depletion. **(E)** The full length of construct contained one important p65 binding motif in Cox2 promoter sequences, and primers for ChIP PCR sequence was designed.


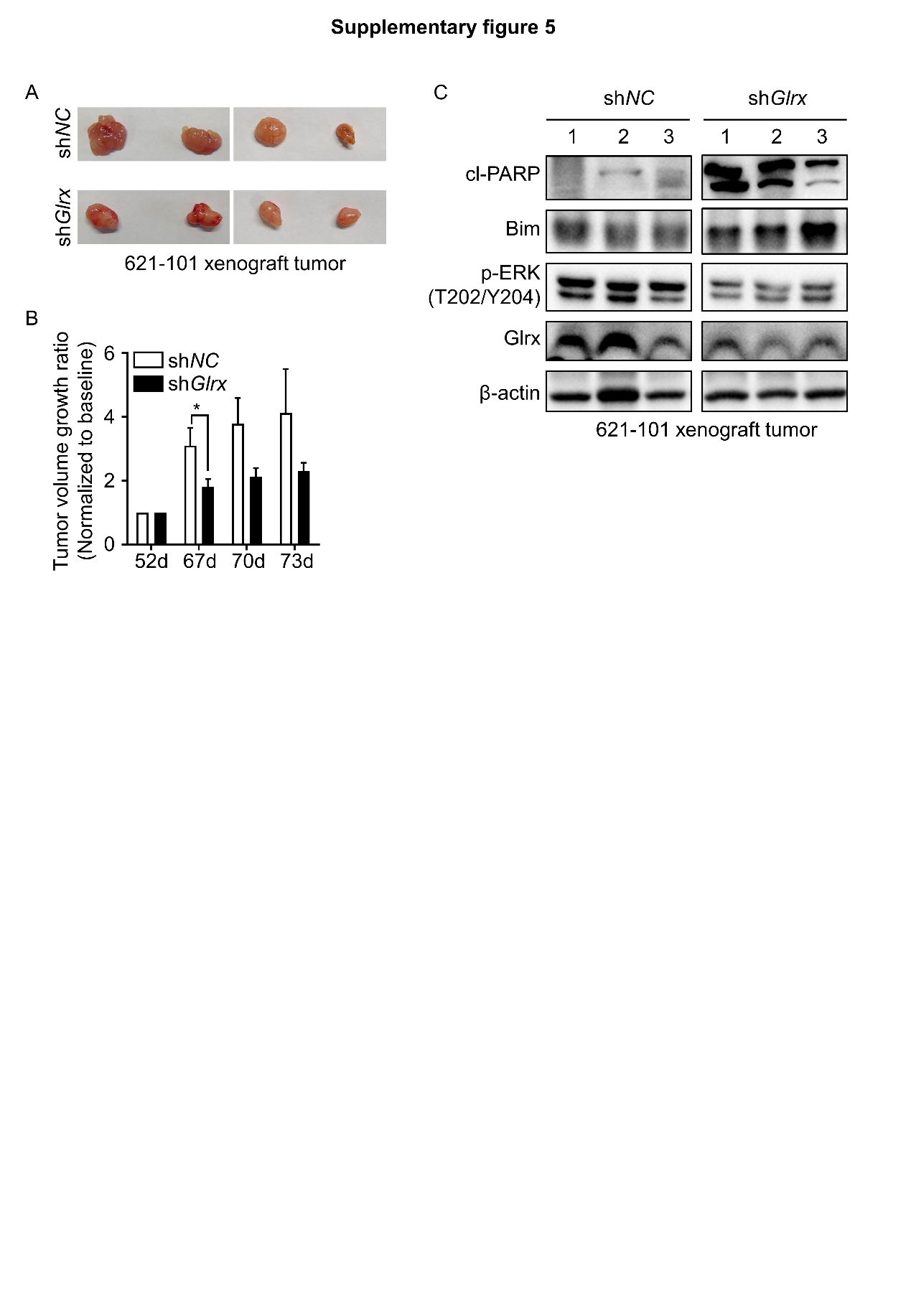


**Supplementary figure 5. Glrx depletion suppressed tumor growth in 621-101 xenograft tumors.** Nude mice were inoculated subcutaneously with 621-101 luciferase-expressing cells with control or Glrx depletion. **(A)** Representative images of tumors were recorded. **(B)** The tumor volume growth ratio was quantified. The tumor volume was calculated using the following formula: V = (L×W^2^)/2. (**C**) Indicated proteins were detected in tumor tissues from groups. Student's t-test, *P < 0.05.
